# Cost-Effectiveness of HPV Self-Testing Options for Cervical Cancer Screening

**DOI:** 10.1001/jamanetworkopen.2025.34960

**Published:** 2025-10-01

**Authors:** Richard T. Meenan, Catherine Lacey, Diana S. M. Buist, Jasmin A. Tiro, John Lin, Melissa L. Anderson, Beverly B. Green, Rachel L. Winer

**Affiliations:** 1 Center for Health Research, Kaiser Permanente Northwest, Portland, Oregon; 2 Department of Epidemiology, University of Washington School of Public Health, Seattle; 3 Kaiser Permanente Washington Health Research Institute, Seattle; 4 Data-driven Strategies for Medicine and Biotechnology, Mercer Island, Washington; 5 Department of Public Health Sciences, University of Chicago Biological Sciences Division, Chicago, Illinois; 6 Washington Permanente Medical Group, Seattle; 7 Department of Health Systems Science, Kaiser Permanente Bernard J. Tyson School of Medicine, Pasadena, California

## Abstract

**Importance:**

Mailing human papillomavirus (HPV) self-sampling kits to underscreened individuals increases cervical cancer screening and can be cost-effective. However, cost-effectiveness has not been evaluated across other screening histories.

**Objective:**

To conduct an economic evaluation of mailed HPV self-sampling among members of a US health care system with adherent, overdue, or unknown screening histories.

**Design, Setting, and Participants:**

This economic evaluation was a cost-effectiveness analysis (CEA) and budget impact analysis (BIA) based on results of a randomized clinical trial (RCT) conducted between November 20, 2020, to July 29, 2022, in an integrated health care system in Washington State. Intervention delivery costs were calculated from Kaiser Permanente Washington and Medicare perspectives and used wellness-based or screening-only visit costs. Participants included female members aged 30 to 64 years identified through electronic medical records. Data were analyzed from August 1, 2022, to July 29, 2025.

**Intervention:**

Members were randomized by screening history. Adherent participants were assigned to 4 groups: usual care (UC), patient reminders, clinician electronic health record [HER] alerts), education (UC and mailed educational materials), direct mail (UC, education, and mailed self-sampling kit), or opt-in (UC, education, and mailed invitation to request kit). Overdue participants were assigned to 3 groups: UC, education, or direct mail. Participants with unknown adherence were assigned to UC, education, or opt-in.

**Main Outcome and Measures:**

Primary RCT outcome was screening completion 6 months postrandomization. CEA outcome was incremental cost-effectiveness ratio for screening completion. BIA outcome was annual program implementation cost over 4 years.

**Results:**

Analyses included 31 355 individuals (mean [SD] age, 45.9 [10.4] years). Among screening adherent members, direct mail dominated all other strategies (more effective and cost-saving). Among overdue members, direct mail was also more effective than UC and generated an additional completed screen at a cost ranging from −$19 (95% CI, −$21 to −$16) (cost saving) to $63 (95% CI, $39 to $87) depending on cost basis and visit type. Among unknown members, opt-in generally dominated UC (more effective and cost-saving). The BIA indicated that although the screening adherent subgroup had the largest year 1 program budget, its budget declined fastest and, by year 4, was lowest among the 3 subgroups. Conversely, the smallest annual budget decreases were among eligible individuals with unknown history.

**Conclusions and Relevance:**

In this economic analysis of a randomized clinical trial, directly mailing HPV kits to individuals who were screening adherent and overdue for screening was economically dominant over other strategies. Program costs declined rapidly over 4 years. Results support directly mailing HPV kits to eligible individuals as an effective, efficient, and affordable outreach strategy.

## Key Points

**Question:**

What is the cost-effectiveness of mailed human papillomavirus (HPV) self-sampling kits (direct-to-all or opt-in) relative to usual care for increasing cervical cancer screening among members of a US health care system with varying screening histories?

**Findings:**

In this economic evaluation of 31 355 participants from a randomized clinical trial, the direct mailing of HPV kits was highly cost-effective among cervical cancer screening adherent and overdue members as was an opt-in approach among members, with unknown screening histories.

**Meaning:**

These findings suggest that within US-based private integrated health care systems, directly mailing HPV kits for cervical cancer screening is cost-effective and affordable.

## Teaser

This economic evaluation examines the cost-effectiveness of mailed human papillomavirus (HPV) self-sampling kits among members of a US health care system with adherent, overdue, or unknown screening histories.

## Introduction

Screening has significantly reduced cervical cancer incidence and mortality,^1^ as most cervical cancers are preventable by identifying and treating high-risk human papillomavirus (HPV) precancers.^2^ In the US, adherence to guideline-recommended screening is declining, from 86% to 73% between 2005 and 2021.^3^ HPV-only (primary HPV) screening can be conducted via home-based testing because, unlike clinic-based Papanicolaou tests, both clinicians and individuals can collect HPV samples.^4^ Home-based testing addresses well-documented screening barriers^5,6^ and can mitigate the impact of appointment backlogs and scheduling constraints on patients and health care systems. Prior US-based^7^ and international research^8^ has focused primarily on the effectiveness and cost-effectiveness of implementing home-based HPV testing in underscreened populations. Findings from the Home-Based Options to Make Cervical Cancer Screening Easy (HOME) trial supported the feasibility^7^ and cost-effectiveness compared with usual care^9^ of mailing HPV kits to individuals overdue for screening.

To generalize the HOME findings beyond underscreened populations, the STEP (Self-Testing options in the Era for Primary HPV screening for cervical cancer) randomized clinical trial was conducted within Kaiser Permanente Washington (KPWA), an integrated health care system in Washington state.^10,11^ STEP evaluated multiple outreach approaches: direct mailing of HPV kits, opt-in to request an HPV kit, and a mailed educational intervention were compared with usual care among populations defined by screening history (screening adherent and now due, overdue, or unknown). Direct mailing of HPV kits increased cervical cancer screening by more than 14% in individuals due or overdue for screening, while opt-in minimally increased screening and education alone did not improve screening rates.^11^ Thus, STEP is the first US-based trial to support the feasibility of mailing HPV kits to screening adherent individuals as an effective strategy for achieving continued adherence.

This study explored the cost-effectiveness of home-based cervical cancer screening interventions among individuals with adherent, nonadherent, and unknown screening histories. This information could inform health care systems on optimal strategies for implementing home-based cervical cancer screening programs. To date, most HPV self-sampling cost-effectiveness research has been conducted in European countries with organized national screening programs.^12,13,14,15,16,17,18^ Although recent trials, including the US, support cost-effectiveness by health care systems,^19,20^ little is known about the cost-effectiveness of home-based HPV testing programs that consider a range of interventions randomized across distinct patient subpopulations. Furthermore, cost-effectiveness analyses have commonly evaluated quality-adjusted life-years (QALYs) rather than more proximal outcomes like completed screens that are more relevant to health care systems to support decision making and resource allocation.^21^ In this economic evaluation, we present a cost-effectiveness analysis of home-based cervical cancer screening interventions based on the STEP trial results,^10,11^ using screening completion as the outcome. Furthermore, our evaluation also includes a budget impact analysis (BIA)^22^ that estimates the overall 4-year cost of implementing a home-based cervical cancer screening program from a US payer perspective to inform budgetary decision-makers.

## Methods

### Settings and Participants

Study design, recruitment details, and results from the STEP clinical trial have been published previously.^10,11^ The KPWA institutional review board (IRB) approved all study procedures for the clinical trial and the economic evaluation. The IRB provided a minimal risk waiver of informed consent for this economic evaluation. This study followed the Consolidated Health Economic Evaluation Reporting Standards (CHEERS) reporting guideline for economic evaluation.^23^

### STEP Trial Intervention and Procedures

Eligible individuals were KPWA members with 3 distinct cervical screening histories: adherent (previously screened, due within 3 months), overdue (Papanicolaou and HPV co-testing more than 5.25 years ago, Papanicolaou testing alone more than 3.25 years ago, or no Papanicolaou testing with minimum continuous KPWA enrollment of 3.25 years), and unknown (KPWA enrollment between 6 months and 3.25 years, no recorded screenings). Inclusion criteria were female sex, age 30 to 64 years, current KPWA enrollment (including assignment to a KPWA primary care clinician), intact cervix, and being due or overdue for cervical cancer screening. Exclusion criteria included electronic health record (EHR) indication of a prior abnormal screen and a nonroutine screening schedule, current pregnancy, or need for an interpreter (kit materials were English-only).

Within each screening history subgroup, individuals were block randomized to usual care (regular mailed patient reminders and clinician EHR alerts), education (usual care and a mailed informational packet including educational materials), direct mail (usual care and educational materials and a mailed HPV self-sampling kit 1 week later), or opt-in (usual care and educational materials with a toll-free number and website link for requesting a self-sampling kit, sent 1 week after request).^10,11^ Overdue individuals were not randomized to opt-in because most international evidence indicated that direct mail was superior for increasing screening.^10^ Individuals with unknown screening history are common in the US, given few screening registries, no universal health care, and freedom to move between systems. Therefore, these individuals were not randomized to direct mail to limit potential overscreening.^11^ If kits were not returned within 3 weeks, study staff made up to 3 reminder calls, consistent with KPWA protocols. Specimens were tested and results documented in the EHR and reported per usual care. Follow-up of HPV-positive results was coordinated through the patient’s primary care clinician and a centralized licensed practical nurse.^10,11^

### Outcomes

The primary STEP trial outcome was screening completion within 6 months after randomization defined as (1) in-clinic screening, (2) kit return with negative or positive HPV 16 or HPV 18 results, or (3) kit return with in-clinic Papanicolaou testing follow-up for other high-risk HPV-positive or unsatisfactory results. Screening completion was assessed using EHR and claims data. Cost-effectiveness was a prespecified secondary objective in the trial protocol, and incremental cost-effectiveness ratio (ICER) for screening completion by outreach approach and prior screening behavior was a prespecified secondary outcome. Focusing on the intermediate outcome of completion, rather than a more distal outcome, such as QALYs, was considered more salient to US-based health care systems and payers. The BIA was not a prespecified analysis. The outcome of the BIA was the estimated 4-year cost of implementing, within a US-based private health care system, such as KPWA, a direct mail screening program among adherent and overdue members or an opt-in program among members with unknown screening history.

### Cost Measurement

Intervention costs, expressed in 2022 US dollars, were defined as the value of resources used to implement the mailed HPV kit program during the trial and were measured from the health care system perspective. Intervention resources were microcosted and classified as labor or nonlabor, including visit type costs, laboratory processing costs by modality (HPV self-sampling kit-based, in-clinic Papanicolaou-based, clinician-collected HPV-based, and co-test screening), and kit outgoing/incoming mailing and component costs (Table 1). Intervention cost data came from the KPWA cost database, the 2022 Centers for Medicare & Medicaid Services (CMS) Physician Fee Schedule,^24^ and actual kit component and mailing costs from the trial. In-clinic screening-related visit costs captured cost differences between private and public payers as well as differences in the clinical context of screening procedures. We specified KPWA-based or CMS-based visit and lab costs and a wellness visit (ie, primary care appointment to manage a comprehensive prevention plan) or a screening-only visit. *Current Procedural Technology* codes were used to develop cost estimates for event-based lab processing and screening visits.^25^

**Table 1.** Intervention Unit Costs (2022 US Dollars)^a^

Intervention unit	Unit cost by cost basis, $	
	KPWA	CMS
Primary care visit		
Age 18-39 y		
Wellness	458.00	160.62
Screening only	209.05	83.36
Age 40-64 y		
Wellness	476.00	167.74
Screening only	209.05	83.36
Mailing costs^b^		
Initial		
Education^c^	1.37	1.37
Direct mail and opt-in^d^	1.93	1.93
Kit		
Outbound^e^	6.23	6.23
Return^f^	2.74	2.74
Processing costs		
Co-testing		
Any normal or unsatisfactory result on Papanicolaou or HPV test	165.00	55.35
Any abnormal result on Papanicolaou or HPV test	228.00	78.39
Primary HPV screening (clinician collected)		
Negative or positive result for HPV-16 and/or HPV-18	113.00	35.09
Other positive result for high-risk HPV (negative result for HPV-16 and HPV-18) and normal or unsatisfactory result on Papanicolaou test	165.00	55.35
Other positive result for high-risk HPV (negative result for HPV-16 and HPV-18) and abnormal result on Papanicolaou test	228.00	78.39
Papanicolaou based screening^g^		
Normal or unsatisfactory	52.00	20.26
LSIL or greater	115.00	43.30
ASCUS and any reflex HPV result	228.00	78.39
HPV test kit screening		
Negative or positive result for HPV-16 and/or HPV-18	113.00	35.09
Unsatisfactory or other positive result for high-risk HPV (negative result for HPV-16 and HPV-18), AND normal or unsatisfactory result on Papanicolaou test	165.00	55.35
Unsatisfactory or other positive result for high-risk HPV (negative result for HPV-16 and HPV-18) AND abnormal result on Papanicolaou test	228.00	78.39

Abbreviations: ASCUS, atypical squamous cells of undetermined significance; CMS, Centers for Medicare & Medicaid Services; HPV, human papillomavirus; KPWA, Kaiser Permanente Washington; LSIL, low grade squamous intraepithelial lesion.

^a^ 2022 intervention unit costs in 2022 US dollars.

^b^ Mailing costs are based on actual research costs in the trial and, therefore, do not vary by KPWA or CMS.

^c^ Includes costs of the envelope, brochure, and postage.

^d^ Includes costs of the envelope, brochure, kit information sheet, and postage.

^e^ Includes costs of the mailer, instructions, HPV self-collect kit, return mailer, and postage.

^f^ Includes costs of the return postage.

^g^ Papanicolaou testing with reflex HPV testing if Papanicolaou is result is either unsatisfactory for review or ASCUS.

### Statistical Analysis

The cost-effectiveness analyses were prespecified in the analysis plan.^10^ The primary economic outcome, ICER for screening completion, was calculated as the difference in cost between 2 groups divided by the difference in number of participants completing screening within 6 months of randomization. Separate ICERs were calculated for the adherent, overdue, and unknown subgroups because of the difference in samples and interventions tested. Inherent uncertainty in the ICERs was assessed by 95% CIs using Fieller theorem.^26,27^ Cost-effectiveness acceptability curves assessing the probability of the comparator condition being cost-effective relative to the baseline condition were calculated at various willingness to pay (WTP) values for an additional completed screening, using the KPWA wellness scenario.

The BIA used parameter values from the underlying STEP clinical trial and costs as described previously to generate total program cost estimates over 4 years for hypothetical target populations of 5000 members each for the screening adherent, overdue, and unknown screening history subgroups. Year 1 screening completion rates were applied for each subsequent year’s remaining eligible individuals. The BIA focused on the direct mail strategy for the adherent and overdue subgroups, and the opt-in strategy for the unknown subgroup. We assumed that only self-sampling kits were sent to health plan members, not educational materials, given the limited effectiveness of such materials in increasing screening rates in the STEP trial.^11^ The BIA focused on screening completion as in the underlying STEP trial (mailed or in-clinic screening). Participants who successfully completed screening were assumed to be removed from the screening pool in future years for either 3 years (Papanicolaou procedure) or 5 years (primary HPV kit or co-testing), based on current guidelines.^28,29,30^ Health plan members entered and exited the 30 to 64 years age range in accordance with the current KPWA age distribution. Per-member per-month cost estimates within each screening subgroup were calculated as the ratio of total costs (divided by 48 total months) to the number of eligible members. As appropriate for BIA, cost estimates were not discounted because of the short time horizon. Data were examined from August 1, 2022, to July 29, 2025. Statistical significance was set at *P* < .05. All analyses used Stata software version 18 (StataCorp).^31^

## Results

### STEP Trial Results

Analyses included 31 355 individuals (mean [SD] age, 45.9 [10.4] years). Between November 2020 and January 2022, randomization was conducted for 13 356 individuals who were screening adherent (40.8%), 8682 who were overdue for screening (26.5%), and 10 733 individuals with unknown screening histories (32.8%). After excluding 1416 randomized in error, 31 355 were included in analyses (13 069 screening adherent [41.4%]; 8311 overdue [26.3%]; 9975 unknown [31.6%]). Baseline characteristics were similar by intervention group within shared screening history.^11^

Prespecified primary analyses for the trial compared direct mail or opt-in individuals with those randomized to the education group. Among the 13 069 individuals who were screening adherent, screening was completed by 914 individuals (61.7%) randomized to direct mail and 2020 (51.1%) randomized to opt-in vs 1885 (47.6%) in the education group. The screening completion absolute difference for direct mail compared with education was 14.1 (95% CI, 11.2 to 16.9) percentage points. For the opt-in group compared with education group, the absolute difference for screening completion was 3.5 (95% CI, 1.2 to 5.7) percentage points. For direct mail compared with opt-in, the absolute difference for screening completion was 10.6 (95% CI, 7.7 to 13.5) percentage points.^11^

Among the 8311 individuals who were overdue, 1408 individuals (35.7%) randomized to direct mail completed screening vs 3506 (18.8%) randomized to education (absolute difference, 16.9 [95% CI, 13.8 to 20.0] percentage points). Among those with an unknown screening history, the 9975 individuals (18.1%) in the opt-in group completed screening vs 3486 (15.9%) in the education group (absolute difference, 2.2 [95.% CI, 0.5 to 3.9] percentage points).

Among the 10 063 individuals who completed screening, 593 (64.9%) returned an HPV self-sampling kit (vs screening in-clinic) in the screening adherent and direct mail group, 430 (21.3%) in the screening adherent and opt-in group, 320 (63.4%) in the overdue and direct mail group, and 113 (17.8%) in the unknown history and opt-in group. Comparisons between education and usual care were not significant, regardless of screening history.^11^

### Economic Results

The unit costs of the STEP trial intervention are shown in Table 1. Total 12-month intervention costs by screening history subgroup are shown in Table 2. Baseline ICERs distinguished by screening history, cost basis (CMS vs KPWA), and visit type (wellness vs screening only) are shown in Table 3. Among members who were screening adherent, the direct mail strategy clearly dominated all other screening strategies (more effective and cost-saving). Among overdue members, direct mail was also more effective than usual care and generated an additional completed screen at a cost ranging from −$19 (95% CI, −$21 to −$16) (cost-saving) to $63 (95% CI, $39 to $87), depending on cost basis and visit type. Among members with an unknown screening history, allowing them to opt into receiving a mailed kit dominated usual care (more effective and cost-saving) in all but the CMS screening-only scenario. Narrow 95% CIs surrounding the ICERs were due to the large sample sizes. eFigures 1 to 3 in Supplement 1 present the cost-effectiveness acceptability curves. Among members who were screening adherent, both direct mail and opt-in had a 100% probability of being cost-effective relative to either usual care or education at a WTP of $0. Among overdue, direct mail had a 97% probability of being cost-effective at a WTP of $0, and a 100% probability at a WTP of $44. For unknown, opt-in had a 74% probability of being cost-effective at a WTP of $0 and reached 100% probability at a WTP of $242.

**Table 2.** Total 12-Month Intervention Costs by Study Group in 2022 US Dollars

Screening history by randomization group	No.	Intervention cost by cost basis and visit type, $			
		KPWA		CMS	
		Wellness	Screening only	Wellness	Screening only
**Adherent**					
Usual Care	3671	686 018	263 369	357 042	214 596
Education	3960	754 024	292 787	394 936	239 493
Opt-in	3956	666 283	270 636	360 970	227 864
Direct Mail	1482	172 879	85 152	108 833	79 252
**Overdue**					
Usual Care	5488	413 875	159 882	215 535	129 829
Education	1408	106 731	42 259	56 426	34 712
Direct Mail	1415	100 905	51 571	64 960	48 342
**Unknown**					
Usual Care	2983	208 742	81 046	108 235	65 717
Education	3486	225 250	90 278	118 959	74 023
Opt-in	3506	220 787	92 620	120 814	78 148

Abbreviations: CMS, Centers for Medicare & Medicaid Services; KPWA, Kaiser Permanente Washington.

**Table 3.** Baseline Incremental Cost-Effectiveness Ratios (ICERs) in 2022 US Dollars

Screening history by cost basis/visit type	ICER (95% CI), $					
	ED to UC	OI to UC	DM to UC	OI to ED	DM to ED	DM to OI
**Adherent**						
KPWA						
Wellness	575 (572-577)^c^	446 (434-457)^a^	461 (336-586)^a^	626 (608-643)^a^	506 (402- 610)^a^	467 (383-551)^a^
Screening only	357 (357-358)^c^	80 (80-80)^a^	91 (79-104)^a^	158 (156-160)^a^	111 (94-128)^a^	96 (88-103)^a^
CMS						
Wellness	402 (401-404)^c^	145 (144-146)^a^	154 (127-181)^a^	242 (238-246)^a^	178 (145- 211)^a^	157 (141-174)^a^
Screening only	329 (329-330)^c^	21 (20-22)^a^	30 (29-30)^a^	83 (82-83)^a^	45 (42-48)^a^	33 (32-33)^a^
**Overdue**						
KPWA						
Wellness	459 (456-462)^b^	NA	19 (16-21)^a^	NA	22 (17-27)^a^	NA
Screening only	753 (752-754)^b^	NA	44 (38-52)^c^	NA	39 (35- 43)^c^	NA
CMS						
Wellness	711 (709-712)^b^	NA	41 (38-45)^c^	NA	35 (35-36)^c^	NA
Screening only	832 (831-832)^b^	NA	63 (39-87)^c^	NA	56 (40-72)^c^	NA
**Unknown**						
KPWA						
Wellness	295 (294-296)^c^	1265 (1265-1266)^a^	NA	53 (51-56)^a^	NA	NA
Screening only	66 (66-67)^c^	95 (95-96)^a^	NA	30 (29-31)^c^	NA	NA
CMS						
Wellness	116 (115-117)^c^	295 (294-296)^a^	NA	24 (22-27)^c^	NA	NA
Screening only	40 (39-40)^c^	95 (95-96)^c^	NA	52 (52-53)^c^	NA	NA

Abbreviations: CMS, Centers for Medicare & Medicaid Services; DM, direct mail; ED, education; KPWA, Kaiser Permanente Washington; NA, not applicable; OI, opt-in; UC, usual care.

^a^ Comparator strategy dominated the baseline strategy (more effective and cost-saving).

^b^ Comparator strategy was dominated by the baseline strategy (less effective and more expensive).

^c^ Comparator strategy was more effective and more expensive than the baseline strategy.

Table 4 and Figure show BIA results for administering the direct mail program to a hypothetical cohort of 5000 health plan members eligible for cervical cancer screening within the adherent and overdue subgroups and the opt-in program within the unknown subgroup. Although the screening adherent subgroup had the largest year 1 program budget, given their highest modeled likelihood of being screened (914/1482 [61.7%]), its budget also declined fastest, and by year 4, was lowest among the 3 subgroups. Conversely, the smallest decreases in annual budgets were within the subgroup with an unknown screening history, which aligned with fewer unknown eligible individuals screened (634/3506 [18.1%]) and exiting the screening pool than those who were adherent or overdue for screening. Within each subgroup the annual program budget varied considerably by cost basis and visit type. For example, within the screening adherent group, the year 1 program budget ranged from $262 236 (CMS and screening-only) to $1 003 707 (KPWA and wellness visit) (Table 4).

**Table 4.** Budget Impact Analysis of Step Mailed HPV Test Program Over 4 Years, By Screening History Subgroup (5000 Eligible Health Plan Members Per Subgroup)^a^

Screening history by randomization group	Program cost by cost base and visit type, $			
	KPWA		CMS	
	Wellness	Screening only	Wellness	Screening only
**Adherent**				
Direct mail				
Year 1	1 003 707	681 154	366 971	262 236
Year 2	369 256	250 591	135 006	96 474
Year 3	128 233	87 024	46 884	33 503
Year 4	149 773	101 642	54 759	39 131
Total	1 650 969	1 120 411	603 619	431 344
PMPM	16.73	11.35	6.12	4.37
**Overdue**				
Direct mail				
Year 1	592 820	405 180	225 888	165 393
Year 2	373 477	255 264	142 309	104 198
Year 3	232 658	159 017	88 652	64 910
Year 4	180 033	123 049	68 600	50 228
Total	1 378 988	942 509	525 449	384 729
PMPM	9.88	6.75	3.76	2.76
**Unknown**				
Opt-in				
Year 1	506 419	319 420	205 146	139 772
Year 2	415 568	257 133	165 143	112 516
Year 3	333 324	206 245	132 460	90 249
Year 4	301 594	186 612	119 851	81 658
Total	1 556 905	969 411	622 599	424 195
PMPM	8.44	5.32	3.42	2.33

Abbreviations: CMS, Centers for Medicare & Medicaid Services; KPWA, Kaiser Permanente Washington; PMPM, per member per month.

^a^ Estimates assume: (1) individuals continuously enrolled for 4 years; (2) does not account for individuals ages 62 to 64 years in year 1 that may age out of screening prior to year 4; (3) age distribution of sample: 18% aged 30 to 39 years and 82% aged 40 to 64 years; (4) proportion of KPWA population aging into eligible population: 0.8%; (5) proportion of KPWA membership aging into eligible population: 2.0%; (6) negative Papanicolaou procedure removes member from screening pool for 3 years; (7) negative HPV test result removes member from screening pool for 5 years; (8) positive HPV-16 or HPV-18 test result permanently removes member from screening pool.

**Figure.**
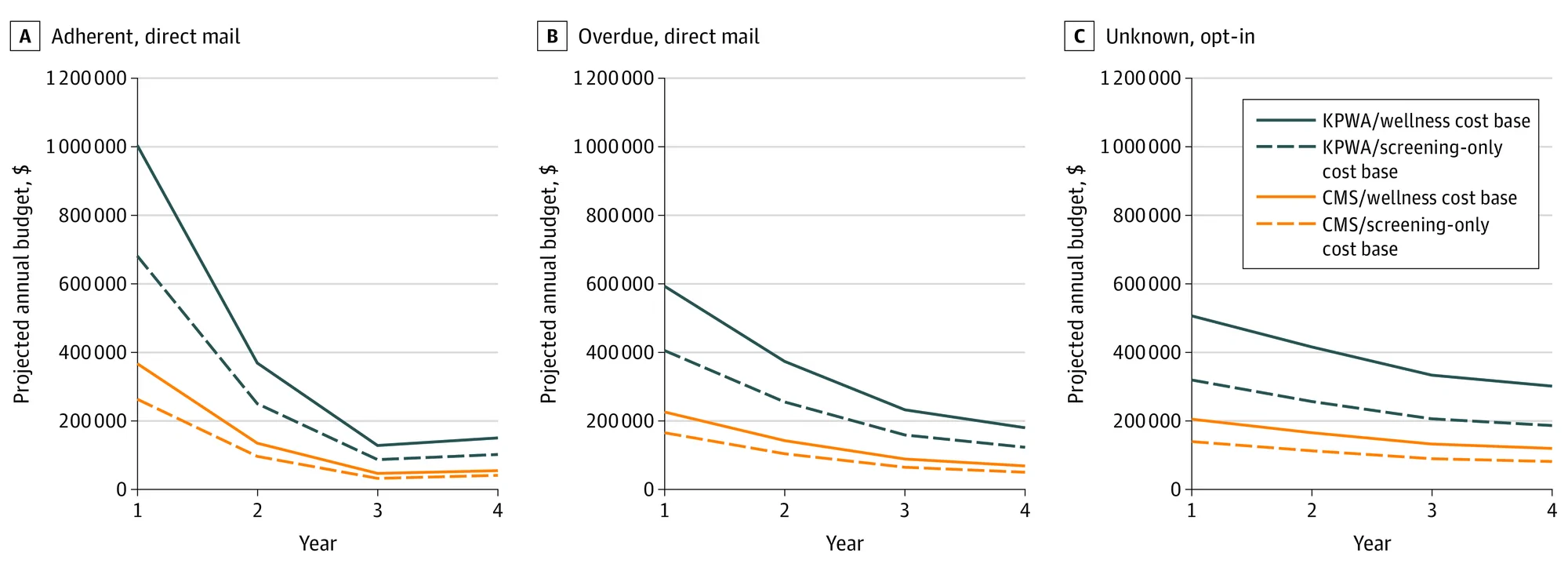
Projected Annual Budget Over 4 Years for STEP Direct Mail Program (5000 Eligible Health Plan Members Per Subgroup)

The per-member per-month (PMPM) program cost (inclusive of in-clinic screening) per screening adherent member (KPWA wellness visit) was $16.73 vs $9.88 for screening-overdue members and $8.44 for members with unknown screening histories. Comparable PMPM values for CMS screening only were as low as $4.37, $2.76, and $2.33, respectively. BIA calculations for the KPWA/wellness scenario are provided in eTables 1 to 3 in Supplement 1.

## Discussion

In this cost-effectiveness analysis of a randomized trial within a US-based integrated health care system, directly mailing HPV self-sampling kits economically dominated other screening strategies among screening adherent members (more effective and cost-saving) and appeared cost-effective among members overdue for screening (more effective, and either cost-saving or with a minimal additional cost per screen). Similarly, inviting members to opt in to receive an HPV kit largely dominated usual care among members with unknown screening histories. However, while opt-in was cost-effective compared with usual care and resulted in cost savings, the absolute increase in screening was small. We also note that our BIA results are broadly comparable to published cost estimates for screening an individual for cervical cancer of $95 (including an office visit) and $32 (without an office visit).^32^

Results are consistent with our previous economic evaluation of the predecessor HOME trial intervention, which focused on increasing cervical cancer screening among underscreened individuals within a US-based private health care system.^9^ Results also provide additional support for our previous systematic review of 16 cost-effectiveness studies of HPV self-collection, which found that HPV self-sampling is cost-effective for increasing cervical cancer screening uptake among underscreened individuals based on findings in 14 of the 16 studies.^12^ In addition, our results are broadly consistent with a recent Danish trial that established the effectiveness of direct mail HPV self-sampling compared with opt-in.^33^

To our knowledge, we conducted the first BIA of a single US-based HPV self-sampling trial. Although a 2019 systematic review^34^ identified 3 BIAs of cervical cancer screening,^35,36,37^ and 2 additional studies from Portugal^38^ and Austria^39^ have since been published, none focused on US settings.

As with our economic evaluation of HOME,^9^ an important distinction of our STEP analysis relative to prior studies is our focus on the intermediate outcome of screening completion, deemed more salient to US-based health care systems and payers^21^ than distal outcomes of cervical cancer diagnosis and treatment or QALYs. Given that health plans—especially those in value-based or Medicare Advantage models—are incentivized by quality performance ratings, our focus on screening completion offers actionable WTP insights for decision-makers allocating resources.

Our analysis benefited from the randomized trial design, combined with a large sample size, which allowed us to evaluate cost-effectiveness within different screening history subgroups. Also, by using both KPWA and CMS cost bases, we were able to compare representative US-based private and public payers. As in the HOME trial analysis,^9^ we included the cost of the underlying clinic visit associated with an in-clinic screening procedure, which we consider an important resource associated with any in-clinic screening performed in conjunction with a mailed HPV testing program. The costs of the mailed program (eg, HPV self-sampling kits, postage) were based on research costs from the trial. We used standard vendors typically used by health care systems and received corporate and/or volume pricing (eg, the USPS corporate priority mail rate), which enhanced the generalizability of the results.

### Limitations

This study has limitations. The trial was conducted within a single US region in a mixed-model health system, where all participants had health insurance and a primary care clinician, which exceeds national averages (86% insured, 75% with a primary care clinician).^40,41^ Additionally, KPWA also uses centralized preventive care reminders. In STEP, HPV-positive results were sent to both primary care clinicians and a centralized licensed practical nurse, likely improving follow-up.^11^ These factors may limit generalizability to other settings, especially to rural or lower-resource settings. Finally, STEP began during the COVID-19 pandemic in late 2020. Cancer screening rates around the world, including for cervical cancer screening, fell substantially during the pandemic due to less interest in and availability of in-person visits. This may have motivated individuals to use HPV self-sampling kits, likely increasing the cost-effectiveness of STEP.^42,43,44^

## Conclusions

In this economic evaluation of various strategies for promoting HPV self-sampling among individuals with different cervical screening histories, directly mailing HPV kits to screening adherent and screening-overdue individuals was economically dominant over other strategies. Direct mail program delivery costs declined rapidly over 4 years. Our results support the direct mailing of HPV self-sampling kits to eligible individuals as an efficient and affordable outreach strategy for raising screening rates in US health systems. Direct mailing may become even more efficient as the use of HPV self-sampling expands in the US, which should be a focus of future economic evaluations.

## Notes

Supplement 1. eTable 1. Budget Impact Analysis For KPWA/Wellness Cost Basis (Adherent Screening History) eTable 2. Budget Impact Analysis For KPWA/Wellness Cost Basis (Overdue Screening History) eTable 3. Budget Impact Analysis For KPWA/Wellness Cost Basis (Unknown Screening History) eFigure 1. Cost-Effectiveness Acceptability Curves For KPWA/Wellness Cost Basis (Adherent Screening History) eFigure 2. Cost-Effectiveness Acceptability Curves For KPWA/Wellness Cost Basis (Overdue Screening History) eFigure 3. Cost-Effectiveness Acceptability Curves For KPWA/Wellness Cost Basis (Unknown Screening History)

Supplement 2. Statistical Analysis Plan and Trial Protocol

Supplement 3. Data Sharing Statement
