# Supplementary material for: Cost-Effectiveness of HPV Self-Testing Options for Cervical Cancer Screening
Source: JAMA Netw Open. 2025 Oct 1;8(10):e2534960. doi: 10.1001/jamanetworkopen.2025.34960 (PMC12489673; doi:10.1001/jamanetworkopen.2025.34960)
Supplement: Supplement 1. — eTable 1. Budget Impact Analysis For KPWA/Wellness Cost Basis (Adherent Screening History) eTable 2. Budget Impact Analysis For KPWA/Wellness Cost Basis (Overdue Screening History) eTable 3. Budget Impact Analysis For KPWA/Wellness Cost Basis (Unknown Screening History) eFigure 1. Cost-Effectiveness Acceptability Curves For KPWA/Wellness Cost Basis (Adherent Screening History) eFigure 2. Cost-Effectiveness Acceptability Curves For KPWA/Wellness Cost Basis (Overdue Screening History) eFigure 3. Cost-Effectiveness Acceptability Curves For KPWA/Wellness Cost Basis (Unknown Screening History) [file jamanetwopen-e2534960-s001.pdf]

Supplemental Online Content

Meenan RT, Lacey C, Buist DSM, et al. An economic evaluation of human Papillomavirus self-testing options for cervical cancer screening. JAMA Netw Open. 2025;8(10):e2534960. doi:10.1001/jamanetworkopen.2025.34960

eTable 1. Budget Impact Analysis For KPWA/Wellness Cost Basis (Adherent Screening History)
eTable 2. Budget Impact Analysis For KPWA/Wellness Cost Basis (Overdue Screening History)
eTable 3. Budget Impact Analysis For KPWA/Wellness Cost Basis (Unknown Screening History)
eFigure 1. Cost-Effectiveness Acceptability Curves For KPWA/Wellness Cost Basis (Adherent Screening History)
eFigure 2. Cost-Effectiveness Acceptability Curves For KPWA/Wellness Cost Basis (Overdue Screening History)
eFigure 3. Cost-Effectiveness Acceptability Curves For KPWA/Wellness Cost Basis (Unknown Screening History)

This supplemental material has been provided by the authors to give readers additional information about their work.

 © 2025 Meenen RT et al. *JAMA Network Open*.

eTable 1. Budget Impact Analysis For KPWA/Wellness Cost Basis (Adherent Screening History)

		Year 1			Year 2			Year 3			Year 4		
Sample	Proportion	N	Population, N	Annual Cost	N	Population, N[a]	Annual Cost	N	Population, N[a]	Annual Cost	N	Population, N[a]	Annual Cost
Beginning mail population[b]			5000	$31,150		1839	$11,460		639	$3,980		746	$4,648
No screening	0.380		-1899			-699			-243			-283	
In-clinic screening	0.217		-1083			-398			-138			-162	
Pap	0.053	57			21			7			9		
Age 30-39	0.010	10		$3,696	4		$1,360	1		$472	2		$551
Age 40-64	0.043	47		$17,178	17		$6,320	6		$2,195	7		$2,563
HPV	0.617	668			246			85			100		
Age 30-39	0.111	120		$46,247	44		$17,014	15		$5,908	18		$6,901
Age 40-64	0.506	548		$214,661	202		$78,972	70		$27,425	82		$32,032
Cotest	0.330	358			132			46			53		
Age 30-39	0.059	64		$26,071	24		$9,591	8		$3,331	10		$3,890
Age 40-64	0.271	293		$134,723	108		$49,564	37		$17,212	44		$20,103
Return HPV kit	0.404		-2018			-742			-258			-301	
HPV neg	0.910	1835		$48,894	675		$17,988	234		$6,247	274		$7,296
16+/18+	0.017	34		$899	12		$331	4		$115	5		$134
Other+/unsat	0.074	148		$60,123	55		$22,119	19		$7,681	22		$8,972
Total annual cost				$583,641			$214,717			$74,566			$87,091
Total 4-year cost				$960,015									
Annual cost per member				$116.73									
Per-member per-month				$9.73									

Abbreviations
KPWA, Kaiser Permanente Washington
HPV, Human Papillomavirus

Footnotes
[a]Estimates assume: 1) individuals continuously enrolled for 4 years; 2) does not account for individuals ages 62-64 years in Year 1 that may age out of screening prior to Year 4; 3) age distribution of sample: 18% age 30-39, 82% age 40-64; 4) proportion of KPWA population aging into eligible population: 0.8%; 5) Proportion of KPWA membership aging into eligible population: 2.0%; 6) Negative Papanicolaou procedure removes member from screening pool for 3 years; 7) negative HPV test result removes member from screening pool for 5 years; 8) positive HPV test result of "16+/18+" permanently removes member from screening pool.
[b]Age distribution of sample: 18% age 30-39, 82% age 40-64

© 2025 Meenen RT et al. *JAMA Network Open.*

eTable 2. Budget Impact Analysis For KPWA/Wellness Cost Basis (Overdue Screening History)

		Year 1			Year 2			Year 3			Year 4		
**Sample**	**Proportion**	**N**	**Population, N**	**Annual Cost**	**N**	**Population, N[a]**	**Annual Cost**	**N**	**Population, N[a]**	**Annual Cost**	**N**	**Population, N[a]**	**Annual Cost**
Beginning mail population[b]			5000	$31,150		3150	$19,625		1962	$12,225		1518	$9,460
No screening	0.642		-3210			-2022			-1260			-975	
						-413							
In-clinic screening	0.131		-655			-398			-257			-199	
Pap	0.097	64			40			25			9		
Age 30-39	0.018	11		$4,106	7		$2,587	5		$1,612	2		$1,247
Age 40-64	0.080	52		$19,087	33		$12,025	21		$7,491	7		$5,796
HPV	0.622	407			257			160			100		
Age 30-39	0.112	73		$28,188	46		$17,758	29		$11,063	18		$8,560
Age 40-64	0.210	334		$130,838	210		$82,428	131		$51,349	82		$39,734
Cotest	0.281	184			116			72			53		
Age 30-39	0.051	33		$13,422	21		$8,456	13		$5,268	10		$4,076
Age 40-64	0.230	151		$69,357	95		$43,695	59		$27,220	44		$21,063
Return HPV kit	0.228		-1140			-718			-447			-346	
HPV neg	0.929	1059		$28,200	667		$17,766	415		$11,068	274		$8,564
16+/18+	0.009	11		$283	7		$178	4		$111	5		$86
Other+/unsat	0.062	71		$28,678	45		$18,067	28		$11,255	22		$8,709
Total annual cost				$353,308			$222,584			$138,659			$107,296
Total 4-year cost				$821,848									
Annual cost per member				$70.66									
Per-member per-month				$5.89									

Abbreviations
KPWA, Kaiser Permanente Washington
HPV, Human Papillomavirus

Footnotes
[a]Estimates assume: 1) individuals continuously enrolled for 4 years; 2) does not account for individuals ages 62-64 years in Year 1 that may age out of screening prior to Year 4; 3) age distribution of sample: 18% age 30-39, 82% age 40-64; 4) proportion of KPWA population aging into eligible population: 0.8%; 5) Proportion of KPWA membership aging into eligible population: 2.0%; 6) Negative Papanicolaou procedure removes member from screening pool for 3 years; 7) negative HPV test result removes member from screening pool for 5 years; 8) positive HPV test result of "16+/18+" permanently removes member from screening pool.
[b]Age distribution of sample: 18% age 30-39, 82% age 40-64

© 2025 Meenen RT et al. *JAMA Network Open.*

eTable 3. Budget Impact Analysis For KPWA/Wellness Cost Basis (Unknown Screening History)

		Year 1			Year 2			Year 3			Year 4		
**Sample**	**Proportion**	**N**	**Population, N**	**Annual Cost**	**N**	**Population, N[a]**	**Annual Cost**	**N**	**Population, N[a]**	**Annual Cost**	**N**	**Population, N[a]**	**Annual Cost**
Beginning mail population[b]			5000	$31,150		4025	$25,076		3228	$20,113		2921	$18,197
No screening	0.817		-4085			-3288			-2638			-2386	
						-413							
In-clinic screening	0.149		-745			-600			-481			-435	
Pap	0.081	60			48			39			35		
Age 30-39	0.015	11		$3,870	9		$3,115	7		$2,499	6		$2,261
Age 40-64	0.066	49		$17,987	40		$14,480	32		$11,614	29		$10,507
HPV	0.562	419			337			271			245		
Age 30-39	0.101	75		$29,005	61		$23,349	49		$18,728	44		$16,944
Age 40-64	0.461	344		$134,633	277		$108,380	222		$86,931	201		$78,647
Cotest	0.357	266			214			172			155		
Age 30-39	0.064	48		$19,390	39		$15,609	31		$12,520	28		$11,327
Age 40-64	0.293	218		$100,195	176		$80,657	141		$64,694	128		$58,530
Return HPV kit	0.034		-170			-137			-110			-99	
HPV neg	0.857	146		$3,882	117		$3,125	94		$2,506	85		$2,268
16+/18+	0.042	7		$190	6		$153	5		$123	4		$111
Other+/unsat	0.101	17		$6,943	14		$5,589	11		$4,483	10		$4,056
Total annual cost				$347,245			$279,533			$224,211			$202,846
Total 4-year cost				$1,053,836									
Annual cost per member				$6,945.00									
Per-member per-month				$5.79									

Abbreviations
KPWA, Kaiser Permanente Washington
HPV, Human Papillomavirus

Footnotes
[a]Estimates assume: 1) individuals continuously enrolled for 4 years; 2) does not account for individuals ages 62-64 years in Year 1 that may age out of screening prior to Year 4; 3) age distribution of sample: 18% age 30-39, 82% age 40-64; 4) proportion of KPWA population aging into eligible population: 0.8%; 5) Proportion of KPWA membership aging into eligible population: 2.0%; 6) Negative Papanicolaou procedure removes member from screening pool for 3 years; 7) negative HPV test result removes member from screening pool for 5 years; 8) positive HPV test result of "16+/18+" permanently removes member from screening pool.
[b]Age distribution of sample: 18% age 30-39, 82% age 40-64

© 2025 Meenen RT et al. *JAMA Network Open.*

eFigure 1. Cost-Effectiveness Acceptability Curves For KPWA/Wellness Cost Basis (Adherent Screening History)

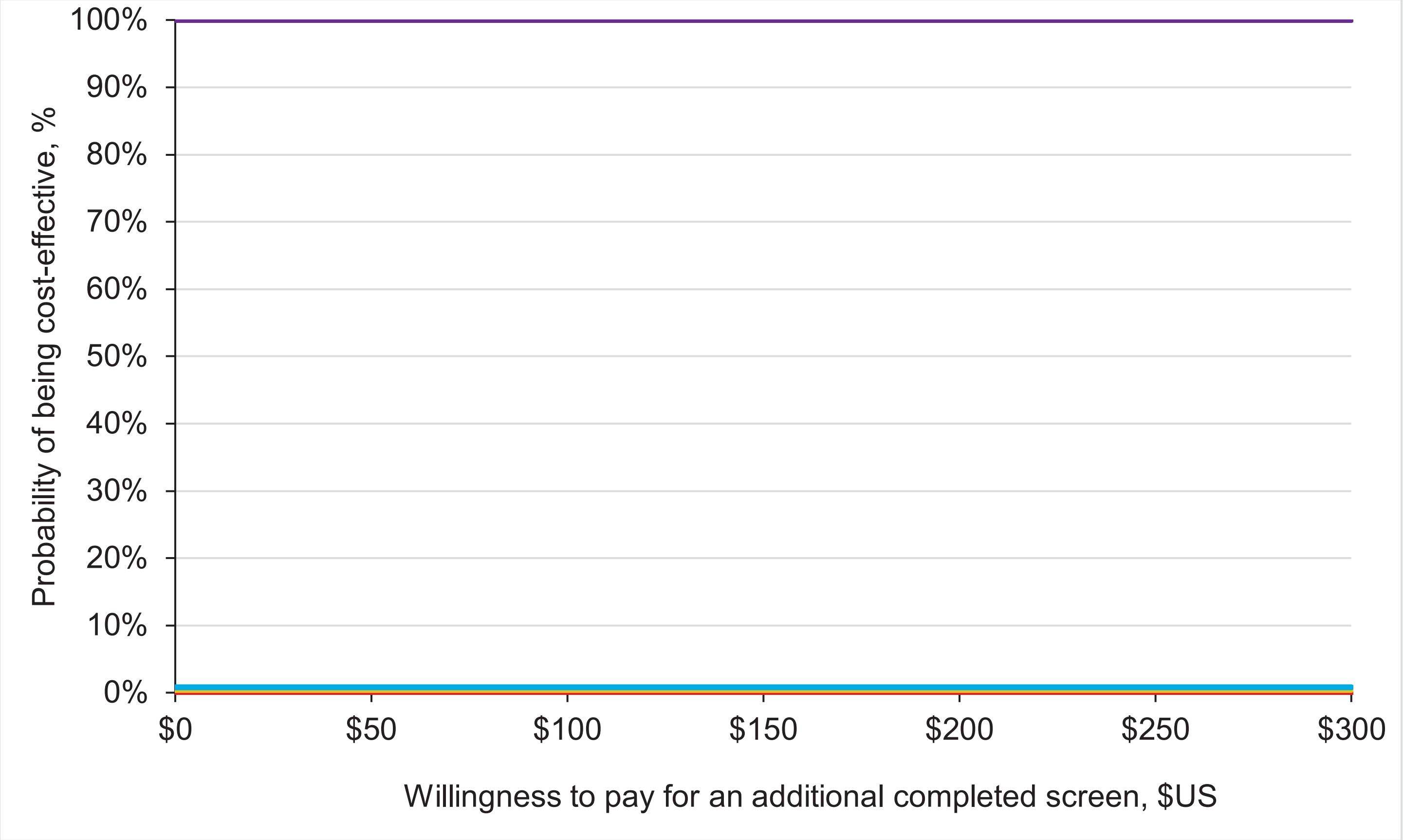

Abbreviations
KPWA, Kaiser Permanente Washington

Legend
— Usual care
— Education
— Opt-in
— Direct mail

 © 2025 Meenen RT et al. *JAMA Network Open.*

eFigure 2. Cost-Effectiveness Acceptability Curves For KPWA/Wellness Cost Basis (Overdue Screening History)

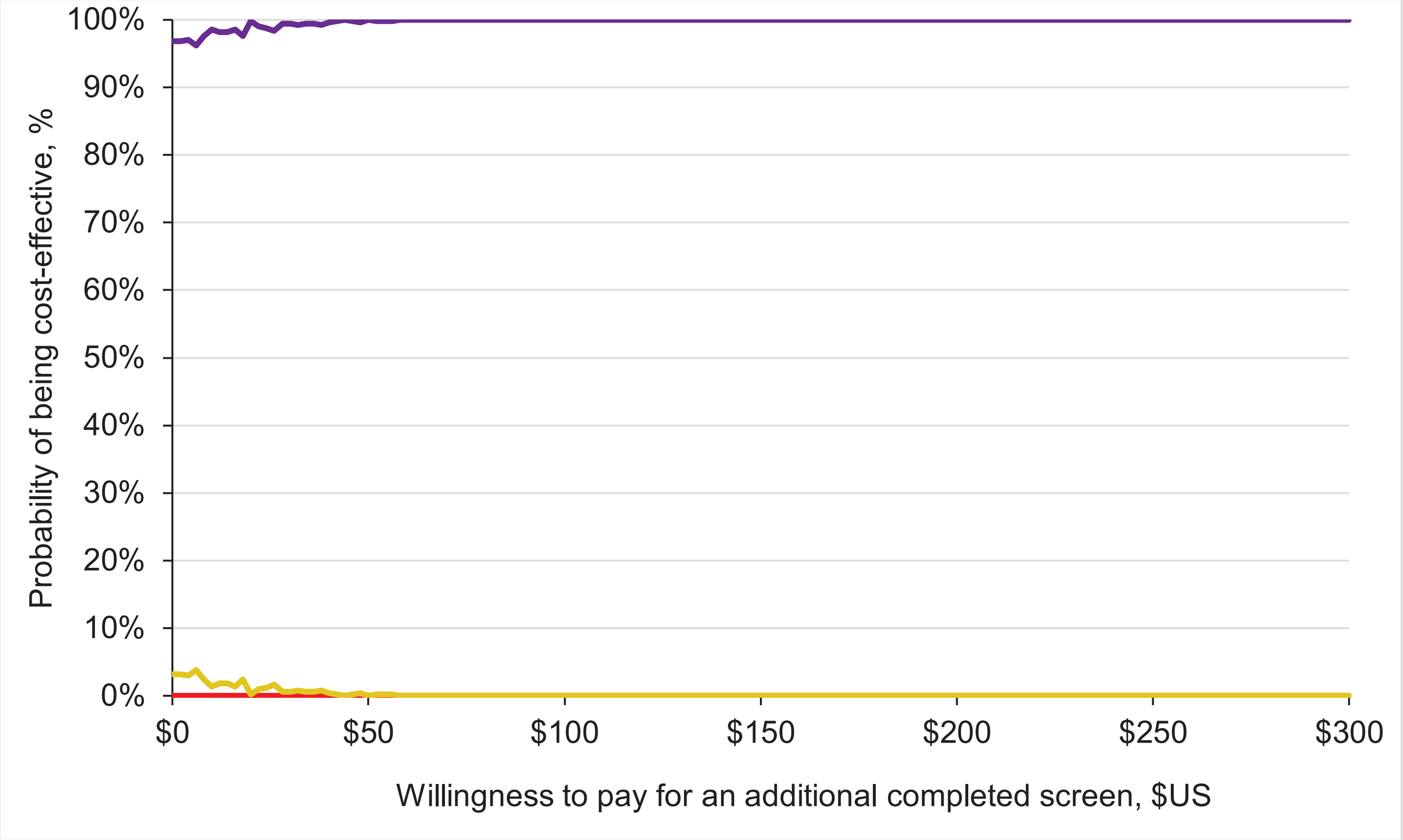

Abbreviations
KPWA, Kaiser Permanente Washington

Legend
— Usual care
— Education
— Direct mail

© 2025 Meenen RT et al. *JAMA Network Open*.

eFigure 3. Cost-Effectiveness Acceptability Curves For KPWA/Wellness Cost Basis (Unknown Screening History)

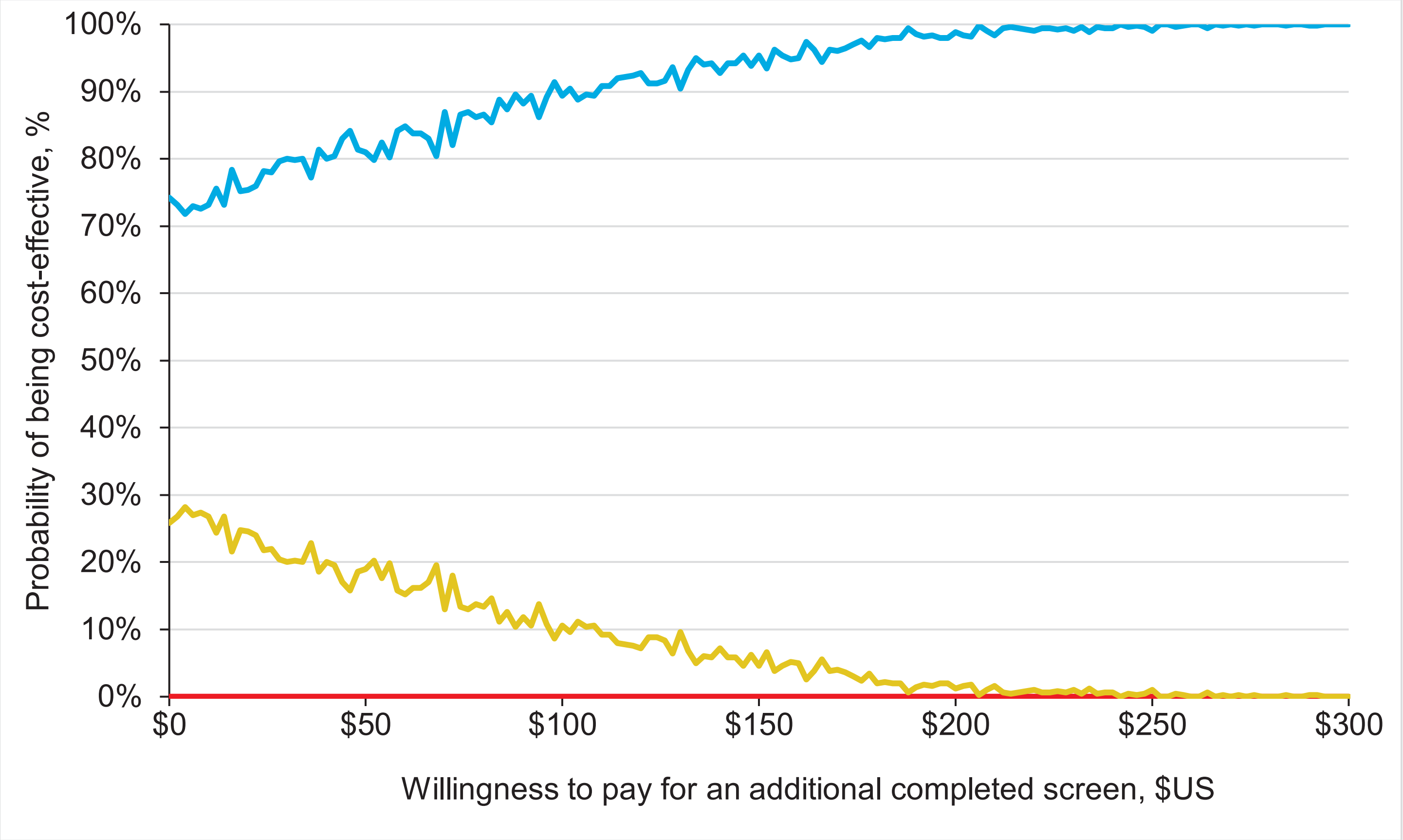

Abbreviations
KPWA, Kaiser Permanente Washington

Legend
— Usual care
— Education
— Opt-in

© 2025 Meenen RT et al. *JAMA Network Open.*
