# Supplementary material for: Cost-Effectiveness of HPV Self-Testing Options for Cervical Cancer Screening
Source: JAMA Netw Open. 2025 Oct 1;8(10):e2534960. doi: 10.1001/jamanetworkopen.2025.34960 (PMC12489673; doi:10.1001/jamanetworkopen.2025.34960)
Supplement: Supplement 2. — Statistical Analysis Plan and Trial Protocol [file jamanetwopen-e2534960-s002.pdf]

ClinicalTrials.gov Identifier:

OFFICIAL TITLE: Self-Testing Options in the era of Primary HPV screening for cervical cancer: the STEP trial

SHORT TITLE: STEP Study

**Coordinating Center:**	Kaiser Permanente Health Research Institute
**Principal Investigators:**	*Diana S.M. Buist (7/2019-2/2022)*<br>*Kaiser Permanente Washington Health Research Institute*<br>*Seattle, Washington, U.S.A.*<br><br>*Beverly B. Green (2/2022-Present)*<br>*Kaiser Permanente Washington Health Research Institute*<br>*Seattle, Washington, U.S.A.*<br><br>*Rachel L. Winer (7/2019-Present)*<br>*Department of Epidemiology*<br>*School of Public Health*<br>*University of Washington*<br>*Seattle, Washington, U.S.A.*
**Study Coordinator:**	*Kris Hansen*<br>*Kaiser Permanente Washington Health Research Institute*<br>*Seattle, Washington, U.S.A.*

Study Exempt from IDE requirements

On 12/16 /2019 (Study Determination request Q191276/S001) the FDA determined the intervention is a nonsignificant risk (NSR) device as it does not meet the definition of a significant risk (SR) device under §812.3(m) of the investigational device exemptions (IDE) regulation (21 CFR 812).

**NCI Award Number:**	R01 CA240375
**ClinicalTrials.gov:**	NCT04679675
**Final Protocol Date:**	9/22/2023
**Protocol Revision Dates:**	12/29/2020<br>8/16/2021<br>9/22/2023
**Initial Protocol Date:**	11/17/2020

Summary of Changes – Protocol

#	Date	Change
1.	12/29/2020	Expand date range of patient history primary objective variables to be captured:<br>- Change cervical cancer screening history interval from 12 months pre-randomization, to most recent screen pre-randomization screen or date of health care system enrollment<br>- Preventative services history from 10 years pre-randomization to 16 years pre-randomization<br>- Add COVID testing/history
2.	8/16/2021	Addition of sub-study of optimized and age-tailored educational brochures.
3.	9/22/2023	Edit 5.3 "Analysis plans" to remove erroneous mention of separate detailed Statistical Analysis Plan (SAP) document.
4.		
5.		

TABLE OF CONTENTS

Summary of Changes – Protocol 2
1. BACKGROUND 4
 1.1 Study Disease(s) 4
 1.2 Rationale 4
2. OBJECTIVES 4
 2.1 Primary Objective 4
 2.2 Secondary Objectives 5
 Schema FIGURE 1: Self-Testing Options in the era of Primary HPV screening for cervical cancer 6
3. PATIENT SELECTION 6
 3.1 Eligibility Criteria 6
 3.2 Exclusion Criteria 6
 3.3 Inclusion of Women and Minorities 6
 3.4 Inclusion of Children 7
4. STUDY PROCEDURES 7
 4.1 Subject Recruitment and Screening 7
 FIGURE 2: STEP Mid-study Educational Brochure allocation for embedded sub-study – optimized and age-targeted 8
 4.2 Procedures 9
 4.3 Early Termination 10
5. STATISTICAL CONSIDERATIONS 10
 5.1 Sample Size 10
 FIGURE 3: STEP randomization allocation by screening history 11
 5.2 Planned Enrollment 12
 5.3 Analysis Plans 12
6. ADVERSE EVENTS: REPORTING REQUIREMENTS 15
 6.1 Determination of Study Risk 15
 6.2 Reporting Adverse Events 15
 6.3 Reporting the Intensity of an Adverse Event 16
 6.4 Reporting the Relationship of an Adverse Event to intervention 16
7. STUDY OVERSIGHT AND DATA REPORTING / REGULATORY REQUIREMENTS 16
 7.1 Protocol Review 16
 7.2 Informed Consent 16
 7.3 Changes to Protocol 17
 7.4 Data and Safety Monitoring Plan 17
8. REFERENCES 17

1. BACKGROUND

1.1 Study Disease(s)

In August 2018, the US Preventive Services Task Force (USPSTF) released updated cervical cancer screening guidelines that include human papillomavirus (HPV) testing alone (i.e., primary HPV screening) as a newly recommended strategy for people with a cervix aged 30-65 years.[1] With primary HPV screening, *home-based screening is an emerging option,* because HPV tests (unlike Pap tests) can be performed on clinician- or self-collected samples. Self-collected samples are as sensitive as clinician-collected samples for detecting HPV and mailing HPV self-sampling kits increases screening participation.[2-6] Australia and the Netherlands—the first countries to implement primary HPV screening—have HPV self-sampling options.[3-7] As US healthcare systems prepare to implement primary HPV screening, they will need to consider a variety of strategies

Our recent pragmatic trial at Kaiser Permanente Washington (KPWA) found that mailing HPV self-sampling kits to overdue individuals increased screening uptake by 50% compared to usual care. Nonetheless, screening remained low in both arms, 30% of HPV-positive individuals did not attend follow-up, and we noted gaps in patient understanding of HPV testing.[8,9] With the USPSTF guideline update,[1] we have a timely opportunity to optimize how health systems *offer* HPV self-sampling outreach to Overdue individuals and test HPV self-sampling outreach approaches among non-Overdue individuals (i.e., those who have successfully screened in the past and are now due). To date, HPV self-sampling randomized clinical trials (RCTs) in the US have included only Overdue individuals[4]; no study has evaluated uptake of HPV self-sampling kits as an alternative to in-clinic screening in a screening-adherent population. Evaluating the cost-effectiveness of different approaches to offering HPV self-sampling kits (e.g., directly mailing kits to all vs. informing individuals about kit availability so they can Opt-In and request the kit) to different health system populations is also an important component of optimizing this strategy.

1.2 Rationale

Pap screening has reduced cervical cancer incidence and mortality by >50% over the last 40 years.[10] Nearly all cervical cancer can be prevented by identifying and removing precancers caused by high-risk HPV. However, US adherence to guideline-recommended screening intervals has declined from a high of 82% in 2003 to 74% in 2016.[11,12] *Increasing screening coverage is a top national priority for cervical cancer prevention*.[1,13] Well-documented barriers include sociodemographic factors (e.g., race and ethnicity and insurance), knowledge about HPV and cervical cancer, beliefs about importance of regular screening, discomfort/embarrassment with the Pap procedure, and scheduling/time barriers.[13-18] Individuals who never or rarely screen are a clear intervention priority, as >50% of the 12,000 cervical cancers diagnosed annually are in people who have not been recently screened.[10,19-21] At the same time, keeping people engaged in routine screening is critical; in 2017, only 48% of KPWA individuals who were up-to-date with Pap testing attended screening within 6 months after their next due date. HPV vaccinations will not end the need for cervical cancer screening,[22] since current vaccines do not protect against all cancer-associated HPV types.[23] Identifying new, patient-centered options that motivate continued screening initiation and completion is essential now and in the future.

2. OBJECTIVES

2.1 Primary Objective

To compare cervical cancer screening completion within six months after randomization among individuals randomized to different outreach approaches, stratified by three categories of prior screening behavior:

A) ***Screening-adherent and coming due:***
Outreach #1: Usual care
Outreach #2: Usual care + Education (hereafter referred to as "Education")
Outreach #3: Usual Care + Education + Opt-In to request an HPV self-sampling kit (hereafter referred to as "Opt-In")
Outreach #4: Usual Care + Education + Direct Mail HPV self-sampling kit (hereafter referred to as "Direct Mail")

B) ***Screen Overdue:***
Outreach #1: Usual care
Outreach #2: Education
Outreach #4: Direct Mail

C) ***Unknown Prior Screening Behavior:***
Outreach #1: Usual care
Outreach #2: Education
Outreach #3: Opt-In

<u>Primary outcome</u>

- Screening completion (*returning the HPV self-sampling kit and attending the recommended in-clinic follow-up screen (if applicable); or receiving in-clinic screening*) within 6 months of randomization

<u>Secondary outcomes</u>

- Screening initiation (*returning the HPV self-sampling kit or receiving in-clinic screening*) within 6 months of randomization
- Time from randomization to screening completion
- Completion of recommended follow-up after a positive HPV self-sampling kit result (per current guidelines, in-clinic Pap after other-HR HPV positive; or colposcopy after HPV 16/18+)
- Initial screening method (none, HPV self-sampling kit, in-clinic)

The main analysis of primary and secondary outcomes will assess differences between Education, Direct Mail and Opt-In, depending on prior screening behavior. Secondary analyses will assess the benefit of education materials alone, by directly comparing Education to Usual Care.

2.2 Secondary Objectives

2.2.1. Evaluate the incremental cost-effectiveness by outreach approach and by prior screening behavior. The outcome will be the incremental cost effectiveness ratio, which will be defined for each outreach approach compared to Education.

2.2.2. Identify patient preference for, and satisfaction with, in-home HPV screening and barriers to follow-up of abnormal screening results (1:1 qualitative interviews and focus groups).

Schema FIGURE 1: Self-Testing Options in the era of Primary HPV screening for cervical cancer

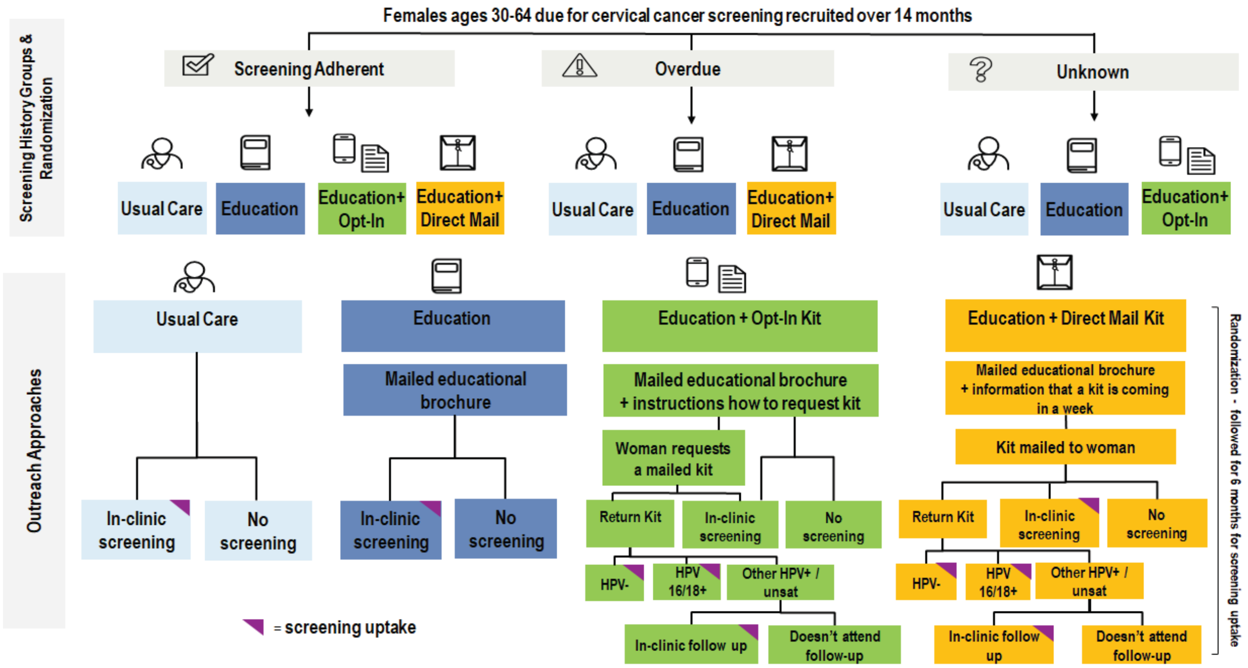

3. PATIENT SELECTION

3.1 Eligibility Criteria

- Currently enrolled at Kaiser Permanente Washington
- Female sex
- 30 years to 64 years of age
- Intact cervix
- Have a primary care provider at Kaiser Permanente Washington
- Due for cervical cancer screening

3.2 Exclusion Criteria

- Flagged by the delivery system as being on a non-routine screening schedule
- Previously randomized to the intervention arm of the HOME trial[24] or invited to the PATH study (P50 CA244432) (participants from the PATH study and individuals randomized to the intervention arm in the HOME trial are excluded from STEP because their prior inclusion in a cervical cancer-related research study could influence their screening uptake and affect the outcome of this trial.)
- On "do not contact list" for research studies
- Currently pregnant or had a pregnancy-related procedure within prior 3 months
- Language interpreter needed

3.3 Inclusion of Women and Minorities

The primary goal of the proposed research is to study whether a programmatic in-home HPV screening outreach strategy improves cervical cancer screening uptake. Because cervical cancer only affects people with a uterine cervix, our entire study population will be composed of female sex individuals. Race and ethnicity are not eligibility requirements for participation in our study. All Kaiser Permanente Washington members who meet our study eligibility requirements will be eligible for random selection

into the study. The ethnic/racial composition of our study population will therefore largely reflect the ethnic/racial composition of Kaiser Permanente Washington female members. The projected proportions of participants from different ethnic/racial backgrounds are based on the composition of Kaiser Permanente Washington members (See Planned Enrollment Report table in Section 5.1).

For the secondary objective (2.2.2) focus groups, we will oversample across non-white race categories to prioritize having at least 60-80% non-white participants overall if possible. For the secondary objective (2.2.2) qualitative interviews, the number of eligible (HPV self-sampling kit positive) individuals is expected to be insufficient to oversample based on race and/or ethnicity. As such, the ethnic/racial composition will largely reflect the ethnic/racial composition of Kaiser Permanente Washington female members.

3.4 Inclusion of Children

We are not enrolling individuals younger than age 30. As of 2020, USPSTF cervical cancer screening guideline recommendations[1] include primary HPV screening only for individuals ages 30 years and older. USPSTF guidelines recommend Pap alone for screening individuals ages 21 to 29. Thus, no participants under age 30 and no children will be included in the study.

4. STUDY PROCEDURES

4.1 Subject Recruitment and Screening

4.1.1 Primary objective (2.1), and secondary objective (2.2.1)

Under a waiver of consent and HIPAA authorization, the study programmer will use electronic medical record data from KPWA to identify individuals who are due for cervical cancer screening based on previous screening history:

1) *Screening-adherent and coming due* (previously screened and due for screening within three months);
2) *Overdue* (never screened; or HPV and Pap co-test >5.25 years ago [or Pap alone >3.25 years ago]; or no past Pap and continuously enrolled at KPWA for ≥3.25 years)*; and
3) *Unknown Prior Screening* (enrolled ≥6 months and <3.25 years, with no recorded screening history).

*Individuals with due dates falling between March 2020-August 2020 will have randomization delayed for 12 months (March 2021-August 2021), because KPWA halted routine cervical cancer screening during these months of the COVID-19 pandemic. A 12-month delay in randomization of these individuals will (1) allow time for the delivery system to catch-up on the 6-month back log of screening visits, and (2) avoid potential misclassification of individuals as overdue solely due to their temporary inability to screen during the halted screening or due to their potential likely difficulty to schedule a screening visit during the expected surge in catch-up visits.

Clinical outreach provided by KPWA includes sending individuals a reminder letter that they are coming due approximately 60 days before their screening due date (for screening-adherent individuals), or repeat reminders every 60 days for individuals who are overdue (known to be overdue, or with an unknown screening history). The study programmer will use these clinical system outreach letters to identify eligible individuals. Each week, we will identify individuals who meet eligibility criteria and are mailed a clinical outreach letter. Eligible individuals will be identified, and a random sample will be randomized to intervention groups, stratified by screening history, as described below. Randomization will occur weekly over a 14-month recruitment period, and eligible individuals will be enrolled under a waiver of consent.

The study programmer will use SAS software built in simple random sample procedure to randomly select study participants from all eligible individuals, and then randomly allocate participants into Usual Care, Education, or 1 of 2 intervention arms based on prior screening history (See Figure 1). *Screening-adherent* individuals will be randomized to one of 4 arms: Usual Care, Education, Opt-In or Direct Mail. *Overdue* individuals will be randomized to Usual Care, Education, or Direct Mail. *Unknown* individuals will be randomized to Usual Care, Education, or Opt-In. Target sample size estimates are detailed in section 5.1. During study enrollment and follow-up, the electronic randomization sequence will be concealed from all researchers by the study programmer, and allocation will only be revealed as minimally necessary to investigators directly involved in data collection and/or safety monitoring.

In March 2021, investigators decided to embed a sub-study to investigate the impact of age-tailored educational materials on screening uptake. The initial wave/week 1-42 educational materials were reviewed and edited via supplemental PATH study grant funding (P50 CA244432). The materials were studied via focus groups conducted by the PATH research group, results were reviewed and brochures edited by the PATH and STEP study teams, and new non-age-specific "optimized"/edited and age-tailored (≤45 and >45 years of age) educational brochures were produced. The sub-study will be implemented from wave/week 43 onward. Individuals randomized to study intervention arms will be stratified by ≤45 versus >45 years of age, and then randomly allocated 1:1 to either age-tailored or non-age-tailored educational materials (see Figure 2). The study programmer will use the same randomization and concealment procedures for the sub-study stratification and allocation.

FIGURE 2: STEP mid-study educational brochure allocation for embedded sub-study – optimized and age-targeted

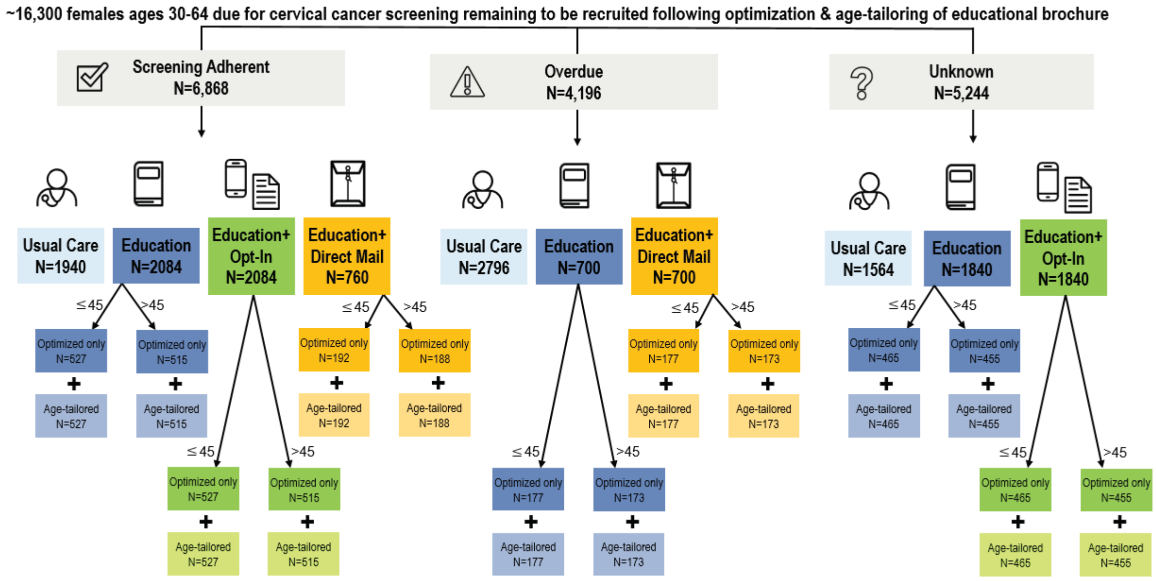

4.1.2 Secondary objective two (2.2.2): Qualitative Interviews

We will conduct a series of in-depth semi-structured 1:1 interviews among participants randomized to the intervention arms of the main study who returned an HPV self-sampling kit that tested HPV positive. We expect to invite approximately 100 – 150 participants and anticipate enrolling up to 50 for interviews. Each week, the study programmer will identify eligible individuals. Participants will be contacted by telephone and those interested in participating will be administered verbal informed

consent under an IRB approved waiver of written consent.

4.1.3 Secondary objective two (2.2.2): Focus groups

We will conduct a series of focus groups among individuals randomized to the intervention arms of the main study who either did not request/return a kit or returned a kit that tested HPV negative. Each week, the study programmer will identify eligible individuals for recruitment. The programmer will oversample from the study population across non-white race categories to prioritize having at least 60-80% non-white participants overall if possible. Participants will be contacted by telephone and those interested in participating will be administered verbal informed consent under an IRB approved waiver of written consent. We will conduct up to eight focus groups of up to 10 people per group to learn about study participant experiences with the educational materials and the HPV self-sampling kits.

4.2 Procedures

4.2.1 Mailings

Individuals randomized to the Usual Care only arm will receive no study materials. Individuals in all other arms will receive the following after randomization:

1. **Introduction letter:** The letter describes the collaboration between our research team and delivery system. For individuals randomized to the Education arm, the letter describes that we are trying to learn how health education materials may help people understand why cervical cancer screening is important. For individuals randomized to the Opt-In or Direct Mail arms, the letter describes that we are trying to learn more about the best ways to offer an in-home cervical cancer screening option as an alternative to screening in a clinic.
2. **Study information sheet:** The study information sheet contains study procedures, potential risks and benefits, measures to protect privacy and confidentiality, and HIPAA compliance. Individuals are informed their participation is voluntary and that they may call a telephone number to “opt-out” of having their individual data included in the research.
3. **Educational Brochure**: Individuals randomized to intervention arms in waves/weeks 1-42 will receive an educational brochure on HPV and cervical cancer that includes information about why screening for cervical cancer is important, key information about HPV and its role in cervical cancer, and different strategies for cervical cancer screening. For the embedded sub-study (wave/week 43 onward), individuals randomized to intervention arms will be stratified by age (≤45 versus >45 years) and then randomly allocated 1:1 to either age-tailored or non-age-tailored educational materials (see Figure 2).
4. **Opt-In or Direct Mail Inserts**: Individuals randomized to the kit arms will receive an informational insert that describes the in-home cervical cancer screening option. Inserts for Opt-In arm participants will additionally include language on how to order a kit.

4.2.2 HPV self-sampling kits

Individuals in the Direct Mail arm will receive their HPV self-sampling kit one week after receiving the above materials. Individuals in the Opt-In arm will receive their HPV self-sampling kit with instructions within a week of requesting a kit. The kit will include a single use COPAN FLOQswab 552C tube with an affixed pre-printed specimen label; biohazard bag with absorbent material; and a postage paid return mailing box. HPV self-sampling kit testing and resulting will follow KPWA’s standard HPV sample processes.

4.2.3 Medical records data
We will obtain data from subject electronic medical records including cervical cancer screening/diagnostic/treatment, medical information related to cervical cancer risk factors, health care use (including preventive services), comorbidities and demographic information.

4.2.4 1:1 Qualitative Interviews
The 1:1 telephone interviews will focus on experiences with follow-up procedures for HPV self-sampling kit intervention arm participants whose results were HPV positive. The 30-minute interview will be recorded and transcribed for coding and thematic analyses. Participants will receive $50 incentive for their participation.

4.2.5 Focus Groups
Focus group discussions will assess participant experiences with the educational material, preferences and satisfaction with the HPV self-sampling kit and barriers to diagnostic testing for those who did not use the kit. A trained facilitator will ask closed-ended items and open-ended questions exploring feelings about self-collection, communication with providers about screenings, and how aspects of the screening process could be more patient-centered. The focus groups will be audio recorded and transcribed for thematic analysis and participants will receive $75 incentive for participation.

4.3 Early Termination
Subjects will not be terminated from the study by the study investigators; however, subjects may choose to decline some or all study procedures. Participants in all arms, except Usual Care only, will be provided a toll-free number that they can call to withdraw consent. All subject withdrawals will be documented in the study database.

5. STATISTICAL CONSIDERATIONS
5.1 Sample Size
Our sample size calculations are based on pairwise comparisons of screening rates and used preliminary data from the KPWA electronic medical record and observed data from the prior HOME trial.[24] Using 2019 KPWA cervical cancer screening data, we estimate 37.1% of screening-adherent, 30.8% of Overdue, and 27.8% of individuals with Unknown screening histories will be screened within 6 months of randomization in the Education arm. Calculations assume a two-sided level 0.05 test, for differences in two proportions.

Our sample size was selected for adequate power to detect meaningful differences in cervical cancer screening rates between outreach strategies within each of the 3 target populations. Meaningful differences were selected based on estimates from the literature[4,25,26] and observed data from the HOME trial.[22] Small improvements in screening uptake are important, particularly if cost-effective and patient-centered; thus, the study is powered to detect relatively small differences between groups. Based on prior studies,[4,25,26] we anticipate the Direct Mail strategy will have a greater impact on screening uptake than the Opt-In approach compared to Education; therefore, we have powered the study for a larger minimal detectable difference for the Direct Mail effect.

We estimated the sample size necessary to have 80% power to detect a 3% difference between Opt-In and Education and a 5% difference in screening completion between Direct Mail and Education. We anticipate that the Direct Mail strategy will have a larger impact on screening outcomes than the Opt-In approach, therefore we selected a larger detectable difference (and thus a smaller sample size needed)

for the Direct Mail group. However, in the screening-adherent population, we computed the sample sizes needed for the 3% and 5% detectable differences respectively, and based the Education arm on the sample size necessary for the Education versus Opt-In comparison, which was larger than necessary for the Education versus Direct Mail comparison. Thus, we will have 80% power to detect a 4.1% difference for the Education vs. Direct Mail screening completion comparison. We selected sample size in the Usual Care group to detect a difference of 3.0% in screening completion between Usual Care and Education for the Screening-Adherent and Unknown populations. Given the smaller sample sizes in the intervention groups for the Overdue population, we were unable to achieve a least detectable difference (LDD) of 3.0% between Usual Care and Education. With a sample size of N=1398 in the Education group (based on an LDD of 3% for Education vs Direct Mail), we computed the detectable difference for a variety of sample sizes for the Usual Care group. We selected a sample size of N=5592 (4 Usual Care participants for each Education participant), because further increasing the Usual Care group led to diminishing improvements in LDD. This sample size yields a least detectable difference of 3.8%.

FIGURE 3: STEP randomization allocation by screening history

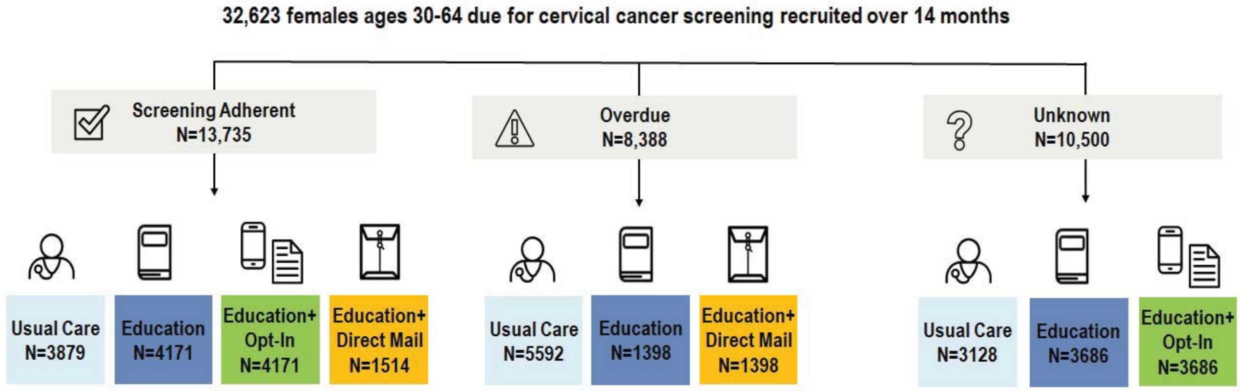

Target sample sizes for each group, stratified by screening history, are shown in Figure 3, and will include randomizing a total of 13,735 Screening-Adherent (3879 Usual Care, 4171 Education, 4171 Opt-In, and 1514 Direct Mail); 8388 Overdue (5592 Usual Care, 1398 Education, and 1398 Direct mail); and 10,500 Unknown (3128 Usual Care, 3686 Education, and 3686 Opt-In).

Study enrollment will occur over a 14-month recruitment period. For all three study populations (Screening-Adherent, Overdue, and Unknown), the number of eligible individuals is estimated to exceed the number needed for the study. Therefore, to reach target sample size over the recruitment period, we will randomly select 231 Screening-Adherent, 142 Overdue, and 1777 Unknown individuals from the pool of all eligible individuals each week, for inclusion in the study. We will allocate each weekly sample to randomization groups as follows: Screening-Adherent (65 Usual Care, 70 Education, 70 Opt-In, and 26 Direct Mail), Overdue (94 Usual Care, 24 Education, and 24 Direct Mail), and Unknown (53 Usual Care, 62 Education, and 62 Opt-In). This sampling strategy maintains a consistent distribution across randomization arms over time, minimizing any potential impacts of temporal trends on group comparisons. If the number of eligible individuals in any given week is lower than the weekly target, the shortfall will be added to the target recruitment sample for the following week.

Beginning with wave 43, each randomization group (except Usual Care) will be further stratified by ≤45 years and >45 years age groups, and then 1:1 randomized to receive age-targeted or non-age-targeted optimized Education brochure as part of their intervention materials (see Figure 2).

Our sample size estimates were based on data from 2019, before the COVID-19 pandemic which has impacted in-person cervical cancer screening. The sample size and recruitment plan described above is our *a priori* sampling strategy to achieve target sample size over the 14-month recruitment period. However, since the number of eligible individuals will vary with each data pull, and may be impacted by changes to care delivery, we will closely monitor recruitment and make necessary changes to ensure target sample sizes are met. Potential changes include the following:

1) If the number of eligible individuals is consistently below the weekly target sample size, we will consider randomizing all eligible individuals rather than a sample, during weeks where there are more eligible individuals than the weekly target. The allocation fractions to randomization groups would remain the same as the original recruitment plan.
2) Since our comparison between Usual Care and Education is an exploratory aim, if the number of eligible individuals is considerably lower than anticipated, we will consider reducing the sample size allocated to Usual Care, to ensure target sample sizes in the Education, Opt-In, and Direct Mail groups are met.

5.2 **Planned Enrollment**

**ANTICIPATED/PLANNED ENROLLMENT for ENTIRE STUDY:**<br>**Number of Participants (*must provide exact numbers. i.e. no range)***			
**Ethnic Categories**	**Sex/Gender**		
	Females	Males	Total
Hispanic or Latino	1,957	0	1,957
Not Hispanic or Latino	30,666	0	30,666
**Ethnic Categories: Total of All Participants**	32,623	0	32,623
**Racial Categories**			
American Indian/Alaska Native	391	0	391
Asian	3,720	0	3,720
Native Hawaiian or Other Pacific Islander	391	0	391
Black or African American	2,120	0	2,120
White	23,717	0	23,717
More Than One Race	2,283		2,283
**Racial Categories: Total of All Participants**	32,623	0	32,623

5.3 Analysis Plans

5.3.1 Analysis plan relevant to primary objectives 2.1 and secondary objective 2.2.1

Figure 1 provides an overview of how our outcomes will be defined by randomization arm.

We define **screening completion** as follows:

<u>Direct Mail or Opt-In</u>:

a) Receive in-clinic screening; or
b) Return kit and complete reflex cytology if recommended per USPSTF primary HPV guidelines.

<u>Usual care or Education</u>:

a) Receiving in-clinic screening.

Screening completion is our primary outcome because of the importance of ensuring individuals follow through with a complete screening episode. One potential disadvantage of a self-collected test is the estimated 8% of individuals who have an other-HR HPV positive or unsatisfactory result[27] who will need to have an in-clinic Pap to determine whether any additional diagnostic evaluation is needed (whereas with in-clinic primary HPV screening, the same sample is used for both HPV testing and reflex cervical cytology).

Screening initiation is a secondary outcome. We define **screening initiation** as either receiving in-clinic cervical cancer screening or returning an HPV self-sampling kit (without the requirement to complete reflex cytology when warranted). Screening initiation is an important indicator of the success or failure of self-collection, since returning an HPV self-sampling kit will count towards cervical cancer screening for Healthcare Effectiveness Data and Information Set quality metrics.[11] If there is a large difference between initiation and completion, it will be informative for implementation efforts (e.g., additional resources may be needed to ensure that individuals with other-HR HPV positive or unsatisfactory kit results complete in-clinic reflex cytology).

All analyses will be stratified by screening history population (Adherent, Overdue, and Unknown). All analyses will be based on the intention to treat principle, with individuals analyzed by the outreach approach arm to which they were randomly assigned. We will first conduct an overall chi-square test to assess differences in observed screening rates among randomization groups. The primary analysis will use a modified Poisson regression modeling approach to estimate the relative risk of screening completion associated with Direct Mail, and Opt-In strategies relative to Education (as appropriate by screening history population). A binary indicator for <u>screening completion</u> within 6 months (yes/no), as defined above, will be the dependent variable. Models will be fit using generalized estimating equations with an independent working correlation structure and robust standard error estimation to account for correlation between participants paneled to the same primary care provider. Models will adjust for baseline characteristics that differ by randomization arm. Models for Overdue participants will also be adjusted for overdue duration (<3 years and ≥3 years). The analysis for the outcome of <u>screening initiation</u> will use the same methods. The primary analysis will not include the Usual Care group, but secondary analyses will include this group, and will estimate the relative risk of screening outcomes for Education, relative to Usual Care.

Delays in screening impact time in compliance with screening recommendations and can also impact cancer outcomes. Thus, our analysis will also assess the time it takes to complete screening, defined as the number of days between randomization and screening completion, as a secondary outcome. We will use Kaplan-Meier log-rank tests to compare time to screening completion by randomization arm, with follow-up time censored at 6 months for non-completers. Analyses will be stratified by screening history population (Adherent, Overdue, and Unknown).

Given the importance of in-clinic follow-up after a positive self-sampling result, data on implementation outcomes will be critical for informing sustainability and translation to other health care systems. We will report the proportion of participants who complete necessary in-clinic follow-up after an abnormal kit result (e.g., reflex cytology after kit result other-HR HPV positive, or diagnostic colposcopy after kit result HPV16/18+) within 6 months after randomization. We will summarize these proportions separately by randomization group (Direct Mail vs. Opt-In) and by prior screening behavior (Adherent, Overdue, and Unknown).

Screening method choice is another important secondary outcome among participants randomized to Opt-In and Direct Mail. We will report the proportion of participants choosing no screening, in-clinic screening, or HPV self-sampling kit. We will summarize these proportions separately by randomization group (Direct Mail vs. Opt-In) and by prior screening behavior (Adherent, Overdue, and Unknown).

Differences in rates of missing data across groups can result in biased estimates of intervention effects. We expect missingness to be minimal since outcome data on screening completion will be defined from automated databases. All randomized participants will be included in the primary analysis, regardless of duration of enrollment in the 6-month follow-up period. In this age group and because outcome assessment is based on only 6 months of post-randomization follow-up, loss to follow-up is expected to be minimal. We do not anticipate any effect of assigned treatment arm on health plan disenrollment. However, we will report the rate of disenrollment by arm. The primary analysis will be unweighted, but if disenrollment rates are high (>10%), we will also perform sensitivity analyses using follow-up duration as an offset parameter in the Poisson regression models. The offset parameter allows estimation of the screening completion rate for censored data where participants had varying lengths of follow-up time.

To protect against multiple comparisons for analyses among the screening-adherent population (which has 3 intervention groups in the primary analysis), we will use the Fisher protected least-significant difference approach, which requires that pairwise group comparisons are made only if the overall omnibus test of any differences between group is statistically significant. The referent group for all analyses will be Education. When evaluating Opt-In and Direct Mail, the primary interest is in the impact of the mailed-kit interventions. The educational materials may also impact screening rates, but by using Education as the referent group for comparisons, the estimated group differences will be the effect related to the mailed kit.

The secondary analysis comparison between Education and Usual Care will allow estimation of the effect of the educational materials alone.

Starting with recruitment wave 43, the original educational brochure was replaced with new brochures, revised based on focus group feedback. A new non-age-specific brochure, plus age-tailored (≤45 and >45 years of age) educational brochures were produced, and study participants, stratified by age group, were randomized to receive the non-age-specific or age-tailored materials. Using data from this embedded sub-study, we will investigate the effects of the revised education materials compared to usual care, and the effects of age-tailored materials compared to non-age-specific materials, on screening completion. This analysis will be limited to individuals randomized to the Usual Care or Education arms, starting with wave 43. We will use a generalized linear model approach, with log link function and Poisson error distribution to estimate the difference in screening completion rates. Indicators for usual care, non-age-specific materials, or age-tailored materials will be the exposure variables, and the binary outcome of screening completion (yes/no) will be the outcome. For the primary analysis, we will assume a constant effect of the revised education materials, and of age-tailoring, across all individuals, and the model will be fit using data from individuals in all screening history groups (Adherent, Overdue, and Unknown), and all ages. In secondary analyses, we will explore whether the effect of the non-age-specific and age-tailored materials differ by screening history or age (≤45 and >45) by adding indicator variables for these characteristics and interaction terms between these variables and the primary exposure variables.

5.3.2 Analysis plans relevant to secondary objective one (2.2.1)

We will conduct a cost-effectiveness analysis of the outreach approaches for in-home HPV self-sampling kits with the incremental cost-effectiveness ratio (ICER) defined as the incremental cost per incremental increase in number of individuals who successfully achieve cervical cancer screening completion as defined by our primary study outcome, which includes appropriate follow-up of other-HR HPV positive HPV self-sampling kit results. The primary economic outcome will be an ICER. For screening-adherent individuals, ICERs will reflect comparisons between a) Opt-In and Education, b) Direct Mail and Education, and c) Direct Mail and Opt-In with Opt-In as the reference strategy. For Overdue individuals, the ICER will compare Direct Mail to Education. For individuals with Unknown screening histories, the ICER will compare Opt-In to Education. Within each screening status group, we will also be able to generate an ICER for Education compared to Usual Care. The study design precludes a comprehensive ICER comparing a single strategy (or set of strategies) to Education or Usual Care across all participants. Costs and screening outcomes will be varied in univariate, multivariable, and/or probabilistic sensitivity analyses as appropriate in Excel-based and Stata-based programs. Discounting and inflation adjustments will not be applied to costs or outcomes because of the 6-month assessment period. The STEP study will allow us to inform the delivery system with screening uptake projections, screening strategy selection, and the relative cost implications of switching from in-clinic to in-home testing, which may differ by prior screening behavior.

5.3.3 Analysis plans relevant to secondary objective two (2.2.2)

We will use data from the interviews and focus groups to evaluate participants' preferences for, and satisfaction with, in-home HPV screening and barriers to follow-up of abnormal screening results. Surveys administered during the interviews and embedded in focus groups discussions will measure knowledge, perceived risk, efficacy beliefs about in-home HPV screening, trust in kit, self-efficacy, kit experience, reasons for not returning kit, screening test preference, future screening intentions, and patient-centered communication around screening.

6. ADVERSE EVENTS: REPORTING REQUIREMENTS

6.1 Determination of Study Risk

This is a minimal risk study where the medical intervention has similar potential adverse events as individuals undergoing standard clinical procedures such as Pap, HPV or other sexually transmitted infection testing.

6.2 Reporting Adverse Events

Based on our own previous and ongoing studies and numerous other prior studies involving self-collected vaginal swabs, we expect adverse events will be rare and minor in severity. The study focus groups and 1:1 qualitative interviews also present minimal risk to subjects as they do not address highly sensitive information. As such, the Principal Investigators and medical monitor will continuously monitor adverse events as they are reported to the study hotline and through individuals' primary care teams. The reviewing institutional review board for this study (Kaiser Permanente Washington) has ruled in agreement with this assessment.

Discomfort and light bleeding are the expected adverse events (AEs). In our previous HOME study of in-home HPV testing, light bleeding was reported by a small minority of subjects who used HPV self-sampling kits (less frequent than bleeding from standard Pap testing). We have no plan for stopping rules due to bleeding because we are only capturing AEs through self-report to the study hotline or primary care teams, and do not expect that the number of reports of bleeding would exceed the

frequency of bleeding from standard Pap testing. All adverse events will be continuously monitored by the Principal Investigators.

All adverse events reported to the study hotline, regardless of causality, will be documented in the electronic adverse event (AE) case report form (CRF). Adverse events will be assessed using the NCI Common Toxicity Criteria for Adverse Events v5.0 (CTCAE). A copy can be downloaded from the CTEP home page:
http://ctep.cancer.gov/protocolDevelopment/electronic_applications/ctc.htm
The Principal Investigators will designate a medical monitor that will be responsible for following serious adverse events (SAEs). The patient should be followed until the event resolves or stabilizes. Frequency of follow-up is at the discretion of the medical monitor. Serious AEs ongoing at the end of the study period must be followed up to resolution.

6.3 Reporting the Intensity of an Adverse Event

Adverse events intensity will be described and graded per NCI Common Terminology Criteria for Adverse Events CTC AE v5.0:
https://ctep.cancer.gov/protocolDevelopment/electronic_applications/docs/CTCAE_v5_Quick_Reference_8.5x11.pdf

6.4 Reporting the Relationship of an Adverse Event to intervention

The medical monitor will assess the causal relationship of the event to study intervention using the following guidelines:
Definite: The event is clearly related to the intervention.
Possible: The event is possibly related to the intervention.
Not related: The event is clearly NOT related to the intervention.
All adverse events, regardless of severity, will be classified as expected or unexpected and reported to the Kaiser Permanente Washington Human Subjects Review Committee, per Kaiser Permanente Washington Human Subjects Review Committee Incident Guidelines, as loaded in IRBNet.

7. STUDY OVERSIGHT AND DATA REPORTING / REGULATORY REQUIREMENTS

7.1 Protocol Review

The protocol and informed consent forms for this study will be reviewed and approved in writing by the Kaiser Permanente Washington Institutional Review Board before any individual is enrolled in this study.

7.2 Informed Consent

7.2.1 Primary objectives and secondary objective one
All consent will be conducted in compliance with Code of Federal Regulations, Title 45, Part 46 (45 CFR part 46). To reduce participation bias, eligible individuals will be identified and randomized into the study under a waiver of consent. Informed consent of Education, Opt-In, and Direct Mail participants will be per a waiver of documentation of consent. These individuals will be notified they have been enrolled in a study and will be provided with the ability to opt-out of having their individual-level medical record data used in the research, but passive consent will be used which will significantly enhance the generalizability of the findings. We will request a waiver of informed consent and a waiver of HIPAA Authorization to identify, enroll and collect data for individuals randomized to Usual Care.

7.2.2 Secondary objective two (2.2.2) (focus groups with participants in Opt-In and Direct Mail arms)
In compliance with 45 CFR part 46, informed consent will be obtained from all participants via verbal consent under a waiver of documentation of consent.

7.3 Changes to Protocol

Any protocol modifications will be approved by the Principal Investigators and approved by the IRB before the revision or amendment may be implemented. The only circumstance in which the amendment may be initiated without regulatory approval is for a change necessary to eliminate an apparent and immediate hazard to the participant. In that event, the investigators will notify the IRB in writing per current IRB rules.

7.4 Data and Safety Monitoring Plan

This is a minimal risk study where the medical intervention has similar potential adverse events as individuals undergoing standard clinical procedures such as Pap, HPV or other sexually transmitted infection testing. There is no data safety and monitoring committee for this study. However, the Principal Investigators will be continuously monitoring adverse events (see Section 6) as they are reported to the study hotline and as reported to the study team by individuals' primary care teams.

Study staff involved in interim data activities will not be involved in any scientific decisions about modifications to the study protocol, but may consult with the scientific leadership team with potential concerns. The scientific leadership team, comprised of the co-Principal Investigators, project senior biostatistician, and one additional co-investigator not involved in data activities, will be blinded to all primary and secondary outcomes review or analyses until 6 months after the last subject is enrolled.

8. REFERENCES

1. U. S. Preventive Services Task Force, Curry SJ, Krist AH, et al. Screening for Cervical Cancer: US Preventive Services Task Force Recommendation Statement. *JAMA.* 2018;320(7):674-686.
2. Winer RL, Feng Q, Hughes JP, et al. Concordance of self-collected and clinician-collected swab samples for detecting human papillomavirus DNA in women 18 to 32 years of age. *Sex Transm Dis.* 2007;34(6):371-377.
3. Arbyn M, Castle PE. Offering Self-Sampling Kits for HPV Testing to Reach Women Who Do Not Attend in the Regular Cervical Cancer Screening Program. *Cancer Epidemiol Biomarkers Prev.* 2015;24(5):769-772.
4. Verdoodt F, Jentschke M, Hillemanns P, Racey CS, Snijders PJ, Arbyn M. Reaching women who do not participate in the regular cervical cancer screening programme by offering self-sampling kits: a systematic review and meta-analysis of randomised trials. *Eur J Cancer.* 2015;51(16):2375-2385.
5. Arbyn M, Verdoodt F, Snijders PJ, et al. Accuracy of human papillomavirus testing on self-collected versus clinician-collected samples: a meta-analysis. *Lancet Oncol.* 2014;15(2):172-183.
6. Leinonen MK, Nieminen P, Lonnberg S, et al. Detection rates of precancerous and cancerous cervical lesions within one screening round of primary human papillomavirus DNA testing: prospective randomised trial in Finland. *BMJ.* 2012;345:e7789.
7. Smith M, Lew JB, Simms K, Canfell K. Impact of HPV sample self-collection for underscreened women in the renewed Cervical Screening Program. *Med J Aust.* 2016;204(5):1941e-1947.
8. Winer RL, Tiro J, Miglioretti D. Can home-based HPV self-sampling improve cervical cancer screening adherence in underscreened women? Results from the HOME pragmatic randomized trial. In. ASPO Abstracts.
9. Winer RL, Tiro J, Gao H. Home-based HPV self-sampling to increase cervical cancer screening participation: A pragmatic randomized trial in a U.S. Healthcare delivery system (Abstract FC 08-05). In.
10. Noone AM, Howlader N, Krapcho M, et al. *SEER Cancer Statistics Review, 1975-2015.* Bethesda, MD: National Cancer Institute.

11. National Committee for Quality Assurance. Cervical Cancer Screening (CCS). https://www.ncqa.org/hedis/measures/cervical-cancer-screening/. Published Published September 7, 2018. Accessed October 19, 2020.
12. National Committee for Quality Assurance. Non-Recommended Cervical Cancer Screening in Adolescent Females (NCS). https://www.ncqa.org/hedis/measures/non-recommended-cervical-cancer-screening-in-adolescent-females/. Published Published September 15, 2018. Accessed October 19, 2020.
13. Saslow D, Solomon D, Lawson HW, et al. American Cancer Society, American Society for Colposcopy and Cervical Pathology, and American Society for Clinical Pathology screening guidelines for the prevention and early detection of cervical cancer. *Am J Clin Pathol.* 2012;137(4):516-542.
14. White A, Thompson TD, White MC, et al. Cancer Screening Test Use - United States, 2015. *MMWR Morbidity and mortality weekly report.* 2017;66(8):201-206.
15. Limmer K, LoBiondo-Wood G, Dains J. Predictors of cervical cancer screening adherence in the United States: a systematic review. *J Adv Pract Oncol.* 2014;5(1):31-41.
16. Zapka J, Taplin SH, Price RA, Cranos C, Yabroff R. Factors in quality care--the case of follow-up to abnormal cancer screening tests--problems in the steps and interfaces of care. *J Natl Cancer Inst Monogr.* 2010;2010(40):58-71.
17. Crawford A, Benard V, King J, Thomas CC. Understanding Barriers to Cervical Cancer Screening in Women With Access to Care, Behavioral Risk Factor Surveillance System, 2014. *Preventing chronic disease.* 2016;13:E154.
18. Akinlotan M, Bolin JN, Helduser J, Ojinnaka C, Lichorad A, McClellan D. Cervical Cancer Screening Barriers and Risk Factor Knowledge Among Uninsured Women. *J Community Health.* 2017;42(4):770-778.
19. Janerich DT, Hadjimichael O, Schwartz PE, et al. The screening histories of women with invasive cervical cancer, Connecticut. *Am J Public Health.* 1995;85(6):791-794.
20. Kinney W, Sung HY, Kearney KA, Miller M, Sawaya G, Hiatt RA. Missed opportunities for cervical cancer screening of HMO members developing invasive cervical cancer (ICC). *Gynecol Oncol.* 1998;71(3):428-430.
21. Leyden WA, Manos MM, Geiger AM, et al. Cervical cancer in women with comprehensive health care access: attributable factors in the screening process. *J Natl Cancer Inst.* 2005;97(9):675-683.
22. Moyer VA, U. S. Preventive Services Task Force. Screening for cervical cancer: U.S. Preventive Services Task Force recommendation statement. *Ann Intern Med.* 2012;156(12):880-891, W312.
23. Markowitz LE. HPV vaccines prophylactic, not therapeutic. *JAMA.* 2007;298(7):805-806.
24. Winer RL, Tiro JA, Miglioretti DL, et al. Rationale and design of the HOME trial: A pragmatic randomized controlled trial of home-based human papillomavirus (HPV) self-sampling for increasing cervical cancer screening uptake and effectiveness in a U.S. healthcare system. *Contemp Clin Trials.* 2018;64:77-87.
25. Kellen E, Benoy I, Vanden Broeck D, et al. A randomized, controlled trial of two strategies of offering the home-based HPV self-sampling test to non- participants in the Flemish cervical cancer screening program. *Int J Cancer.* 2018;143(4):861-868.
26. Tranberg M, Bech BH, Blaakaer J, Jensen JS, Svanholm H, Andersen B. Preventing cervical cancer using HPV self-sampling: direct mailing of test-kits increases screening participation more than timely opt-in procedures - a randomized controlled trial. *BMC Cancer.* 2018;18(1):273.
27. Winer RL, Lin J, Tiro JA, et al. Effect of Mailed Human Papillomavirus Test Kits vs Usual Care Reminders on Cervical Cancer Screening Uptake, Precancer Detection, and Treatment: A Randomized Clinical Trial. *JAMA Netw Open.* 2019;2(11):e1914729.
