# Supplementary material for: Cost-Effectiveness of HPV Self-Testing Options for Cervical Cancer Screening
Source: JAMA Netw Open. 2025 Oct 1;8(10):e2534960. doi: 10.1001/jamanetworkopen.2025.34960 (PMC12489673; doi:10.1001/jamanetworkopen.2025.34960)
Supplement: Supplement 3. — Data Sharing Statement [file jamanetwopen-e2534960-s003.pdf]

Data Sharing Statement

Data
Data available: Yes
Data types: Deidentified participant data, Data dictionary
How to access data: Requests for data must be sent to rlw@uw.edu
When available: With publication

Supporting Documents
Document types: None

Additional Information
Who can access the data: Data will be made available to researchers whose proposed use of the data has been approved.
Types of analyses: Data will be made available for a specified purpose.
Mechanisms of data availability: Data will be made available without investigator support to researchers with adequate resources to cover the regulatory and data sharing costs. Data will be made available after approval of a concept proposal aligned with current data approvals, and with a signed data access agreement.
